# Kv1.5 channel mediates monosodium urate-induced activation of NLRP3 inflammasome in macrophages and arrhythmogenic effects of urate on cardiomyocytes

**DOI:** 10.1007/s11033-022-07378-1

**Published:** 2022-04-04

**Authors:** Peili Li, Yasutaka Kurata, Fikri Taufiq, Masanari Kuwabara, Haruaki Ninomiya, Katsumi Higaki, Motokazu Tsuneto, Yasuaki Shirayoshi, Miguel A. Lanaspa, Ichiro Hisatome

**Affiliations:** 1 Department of Genetic Medicine and Regenerative Therapeutics, Institute of Regenerative Medicine and Biofunction, Tottori University, 36-1, Nishimachi, Yonago, Tottori 683-8504 Japan; 2 Department of Physiology II, Kanazawa Medical University, Kahoku, Ishikawa 920-0293 Japan; 3 Intensive Care Unit and Department of Cardiology, Toranomon Hospital, Tokyo, 105-8470 Japan; 4 Department of Biological Regulation, Tottori University, Yonago, 683-8504 Japan; 5 Research Center for Bioscience and Technology, Tottori University, Yonago, 683-8504 Japan; 6 Division of Renal Diseases and Hypertension, School of Medicine, University of Colorado Denver, Aurora, CO 80045 USA

**Keywords:** Monosodium urate, Kv1.5, NLRP3 inflammasome, Atrial myocytes

## Abstract

**Background:**

Gout is usually found in patients with atrial fibrillation (AF). K+ efflux is a common trigger of NLRP3 inflammasome activation which is involved in the pathogenesis of AF. We investigated the role of the K+ channel Kv1.5 in monosodium urate crystal (MSU)-induced activation of the NLRP3 inflammasome and electrical remodeling in mouse and human macrophages J774.1 and THP-1, and mouse atrial myocytes HL-1.

**Methods and Results:**

Macrophages, primed with lipopolysaccharide (LPS), were stimulated by MSU. HL-1 cells were incubated with the conditioned medium (CM) from MSU-stimulated macrophages. Western blot, ELISA and patch clamp were used. MSU induced caspase-1 expression in LPS-primed J774.1 cells and IL-1β secretion, suggesting NLRP3 inflammasome activation. A selective Kv1.5 inhibitor, diphenyl phosphine oxide-1 (DPO-1), and siRNAs against Kv1.5 suppressed the levels of caspase-1 and IL-1β. MSU reduced intracellular K^+^ concentration which was prevented by DPO-1 and siRNAs against Kv1.5. MSU increased expression of Hsp70, and Kv1.5 on the plasma membrane. siRNAs against Hsp70 were suppressed but heat shock increased the expression of Hsp70, caspase-1, IL-1β, and Kv1.5 in MSU-stimulated J774.1 cells. The CM from MSU-stimulated macrophages enhanced the expression of caspase-1, IL-1β and Kv1.5 with increased Kv1.5-mediated currents that shortened action potential duration in HL-1 cells. These responses were abolished by DPO-1 and a siRNA against Kv1.5.

**Conclusions:**

Kv1.5 regulates MSU-induced activation of NLRP3 inflammasome in macrophages. MSUrelated activation of NLRP3 inflammasome and electrical remodeling in HL-1 cells are via macrophages. Kv1.5 may have therapeutic value for diseases related to gout-induced activation of the NLRP3 inflammsome, including AF.

**Supplementary Information:**

The online version contains supplementary material available at 10.1007/s11033-022-07378-1.

## Introduction

Gout is the most common form of inflammatory arthritis, affecting 3–6% of men and 1–2% of women in developed countries. Gout is associated with hyperuricemia [1]. The ionized forms of uric acid (UA) form monosodium urate (MSU). MSU deposits in joints and surrounding tissues are uptaken by macrophages, causing inflammation via activation of NLRP3 (nucleotide-binding domain, leucine-rich repeats, and pyrin domain-containing protein 3) inflammasome, a cytosolic multiprotein platform [2].

The NLRP3 inflammasome is a major signaling pathway of the innate immune system, and is associated with gout, atherosclerosis, atrial fibrillation (AF), and other diseases [3, 4]. Upon activation by stimulatory particles, such as MSU or cholesterol crystals, NLRP3 interacts with the adaptor protein ASC (apoptosis-associated speck-like protein containing a caspase-1 recruitment domain) and pro-caspase-1 to generate bioactive capsase-1(p20) [2]. Cleavage of precursor pro-IL-1β by capsase-1 yields active IL-1β (p17). Phagocytosis of particulate matters induces K^+^ efflux, thus reducing cytosolic K^+^. K^+^ efflux ultimately serves as an upstream requirement to activate the NLRP3 inflammasome [5]. However, the mechanism underlying K^+^ efflux remains unknown.

Kv1.5 channels, encoded by *KCNA5*, confer ultra-rapid delayed-rectifier potassium outward current (*I*_Kur_). Kv1.5 is expressed in murine atrial myocytes and in macrophages [6, 7]. Uric acid enhances Kv1.5 protein expression in HL-1 mouse atrial myocytes via post-transcriptional modification by Hsp70, resulting in increases of the channel function [8]. Hyperuricemia and gout are related to the incidence of paroxysmal or persistent AF [9, 10]. Excess NLRP3 signaling in atrial myocytes augments Kv1.5 expression and *I*_Kur_, which results in abbreviated atrial effective refractory period and enhanced AF susceptibility [4].

The purpose of this study was to clarify the role of Kv1.5 channel in MSU-induced activation of NLRP3 inflammasome in macrophages and electrical remodeling in atrial myocytes.

## Materials and methods

### Cell culture and channel treatments

Mouse macrophage cell line J774.1 and human macrophage cell line THP-1 (JCRB cell bank) were cultured in RPMI medium supplemented with 10% fetal bovine serum at 37 °C in 5% CO_2_. HL-1 mouse cardiomyocytes were cultured in Claycomb medium (Sigma Aldrich) as previously described [6]. The macrophages were plated into 6-well tissue culture plates (1.5 X10^6^ cells per well in 2 ml medium) overnight then primed with 1 μg/ml lipopolysaccharide (LPS) (from Sigma) in 1 ml serum-free RPMI medium for 6 h. After priming, the culture medium was replaced with 1 ml OPTI-MEM (Life Technologies) containing MSU (500 μg/ml) (Nacalai Tesque, Inc) for 6 h. K^+^ channel inhibitors or the inflammasome inhibitor MCC950 (50 nM) (Cayman Chemical Co.) were added to OPTI-MEM 30 min prior to the addition of MSU (Nacalai Tesque, INC). The following K^+^ channel inhibitors were used: non-selective voltage-gated K^+^ channel inhibitor, 4-aminopyridine (4-AP) (6 mM) (Tokyo Chemical Industry Co, LTD); a selective Kv1.5 channel inhibitor, diphenyl phosphine oxide-1 (DPO-1) (1 μM) (R&D systems); a selective Kv1.3 channel inhibitor, 5-(4-phenoxybutoxy) psoralen (PAP-1) (50 nM) (Chemscene LLC); a TWIK2 K^+^ channel blocker, quinine (1 μM) (Fuji Film, Japan); an inward-rectifier K^+^ channel blocker, BaCL_2_ (0.1 mM, 1 mM) (Fuji Film, Japan); and a ATP-sensitive K^+^ channel inhibitor, glibenclamide (1 μM) (Bio Vision, Milpitas, CA, USA). The conditioned medium (CM) from untreated J774.1 cells or LPS-primed and MSU-treated cells was added to 6 X 10^5^ HL-1 cells in 6-well plates overnight.

### Immunoblot analysis

Cells were harvested and lysed by sonication in a buffer (PBS supplemented with 1% polyoxyethylene octyiphenyl ether (NP-40), 0.5% sodium deoxycholate, 0.1% SDS, 1.5 mM aprotinin, 21 mM leupeptine, 15 mM pepstain, and 1 mM phenylmethylsulfonyl fluoride). After removal of insoluble materials by centrifugation, protein concentrations were determined using a bicinchoninic acid (BCA) protein assay kit (Pierce, Biotechnology, Rockford, IL, USA). Culture supernatants were collected after inflammasome stimulation by MSU. The proteins in the supernatants were concentrated using StrataClean resin (Agilent Technologies, Santa Clara, CA, Cat# 400714) according to the manufacturer’s instructions. Bound proteins were recovered by incubation at 95 °C for 5 min in a sample buffer. Proteins were separated by SDS-PAGE and electrotransferred to PVDF membranes. The membranes were blocked with 5% non-fat dry milk in PBS containing 0.1% Tween and were subsequently subjected to immunoblot (IB) analysis with a primary antibody. The antibodies used were as follows: NLRP3 (Cell Signaling, Cat# 15101), ASC (AdipoGen, Cat# AG-25B-0006) and caspase-1 (AdipoGen, Cat# AG-20B-0042, AG-20B-0048), IL-1β (R& D systems, Cat# AF-401-NA), Kv1.5 and Kv1.3 (alomone Labs, Cat# APC-004, APC-002), Na^+^/K^+^ ATPase α-1 (upstate, Cat# 05-369), Hsp70 (Stressgen, Cat# SPA-810), and β-actin (Calbiochem, Cat# CP01). The blots were developed using an ECL system (Amersham, Biosciences, Piscataway, NJ, USA) and band densities were quantified using NIH Image J software. For the chase experiment, J774.1 cells (1.5 X10^6^ cells per well in 2 ml medium) were passaged into 6-well culture plates. cycloheximide (60 μg/ml) (Fujifilm, JP) was added to the culture medium after the LPS-primed cells were stimulated with MSU for 6 h. Cells were harvested at the indicated time points then analyzed by IB.

### Subcellular fractionation

Cell fractionation was performed as described previously [11]. LPS-primed cells were treated with MSU for 6 h. Lysates from LPS-primed MSU-treated cells and control cells (without LPS and MSU treatments) were obtained and separated into cytosolic and plasma membrane fractions using the Plasma Membrane Protein Extraction Kit (BioVision, Milpitas, CA, USA, Cat# K268-50) according to the manufacturer’s instructions. Each fraction was subjected to 7.5% SDS-PAGE and analyzed by IB. Na^+^/K^+^-ATPase and β-actin were used as markers of the plasma membrane and cytosolic subcellular fraction, respectively.

### RNA-mediated interference

siRNA transfection into J774.1 and HL-1 cells was performed using Lipofectamine RNAiMAX (Invitrogen, Cat# 13778150) following the manufacturer’s protocols. Two pairs of siRNAs that target Kv1.5 and Kv1.3 were used, along with an siRNA against Hsp70 and a scrambled control siRNA. Table 1 shows sequences of siRNAs.

**Table 1:** Sequences of siRNA

	Sense	Antisense
Kv1.5-1	5′ UCA UCA AGG AAG AGG AGA AUU 3′	5′ UUC UCC UCU UCC UUG AUG AUU 3′
Kv1.5-2	5′ ACC UAA AGG CCA AGA GCA AUU 3′	5′ UUG CUC UUG GCC UUU AGG UUU 3′
Kv1.3-1	5′ GGA CAG ACG CUG AAG GCU UUU 3′	5′ AAG CCU UCA GCG UCU GUC CUU 3′
Kv1.3-2	5′ CCA GAA AUA UCA UGA ACU UUU 3	5′ AAG UUC AUG AUA UUU CUG GUU 3′
Hsp70-1	5′ CUG GAG AUC GAC UCU CUG UUC 3′	5′ ACA GAG AGU CGA UCU CCA GGC 3′
Scramble	5′ GAA GCG AGA UAU CCC UGA CTT 3′	5′ GUC AGG GAU AUC UCG CUU CTT 3′

### Immunofluorescence

Cells were seeded at 1 × 10^5^ per slide onto gelatin-coated coverslips in a 3.5 cm dish. After LPS-priming, DPO-1 (1 μM) was applied to the cells 30 min prior to MSU stimulation for 6 h. The cells were fixed with 4% paraformaldehyde/PBS and permeabilized with 0.5% Triton X-100. The cells were then incubated for 1 h at room temperature with a primary antibody (ASC, 1:200, Enzo, Cat# ADI-905-173). After blocking in 3% albumin, bound antibodies were visualized with an Alexa Fluor 488-conjugated rabbit secondary antibody (1:1000) (ThermoFisher scientific, Cat# A32731). Images were obtained using a Bio-Rad MRC 1024 confocal microscope.

### ELISA

IL-1 β concentrations in culture supernatants of J774.1 or THP-1 cells, and HL-1 cell lysates were measured by ELISA according to the manufacturer’s instructions (R& D System, cat#: MLB00C and DLB50).

### Measurement of intracellular potassium concentration

The intracellular K^+^ concentration of J774.1 cells was measured using the FluxOR™ potassium ion channel assay (Life Technologies, Cat# F10017) as previously described [12]. Briefly, J774.1 cells were seeded in 6-well plates and primed with LPS (1 μg/ml) for 6 h. The LPS-primed cells were treated with MSU (500 μg/ml) for 1 h to prevent the cell death. Fluorescence was measured at an excitation wavelength of 480 nm and an emission wavelength of 530 nm.

### Electrophysiological recordings

Kv1.5-mediated currents corresponding to *I*_kur_ in J774.1 and HL-1 cells were measured at 37 °C using whole-cell patch-clamp techniques with an Axopatch-200 amplifier (Axon Instruments, USA). The procedures for the current measurement were essentially the same as described previously [6]. The extracellular solution contained (mM): NaCl 140, KCl 4, CaCl_2_ 1.8, MgCl_2_ 0.53, NaH_2_PO_4_ 0.33, glucose 5.5, and HEPES 5, with a pH adjusted to 7.4. The internal pipette solution contained (mM) K-aspartate 100, KCl 20, CaCl_2_ 1_,_ Mg-ATP 5, EGTA 5, HEPES 5, and creatine phosphate dipotassium salt 5 (pH 7.2 with KOH). The currents were elicited every 6-s by 500-ms test pulses ranging from − 60 to + 40 mV ( in 10 mV increments) with a holding potential of − 60 mV. DPO-1 (1 μM) was added to the bath solution to block Kv1.5 channel currents. The DPO-1 sensitive currents were obtained by digitally subtracting the current traces in the presence of DPO-1 from those in the absence of DPO-1. Action potentials (APs) of HL-1 cells were elicited at 0.5 Hz by 5-ms square current pulses of 1nA, and sampled at 20 kHz. AP durations (APDs) were measured at 20%, 50% and 90% repolarization (APD_20_, APD_50_ and APD_90_), respectively.

### Statistical analysis

All data are presented as the mean ± SEM. Student’s *t* test and repeated measures analysis of variance (two-way ANOVA) were used for comparisons of two and multiple (more than two) groups, with *p* values < 0.05 considered statistically significant.

## Results

### Kv1.5 channel is required for MSU-induced NLRP3 inflammasome activation in J774.1 cells

**Fig. 1:**
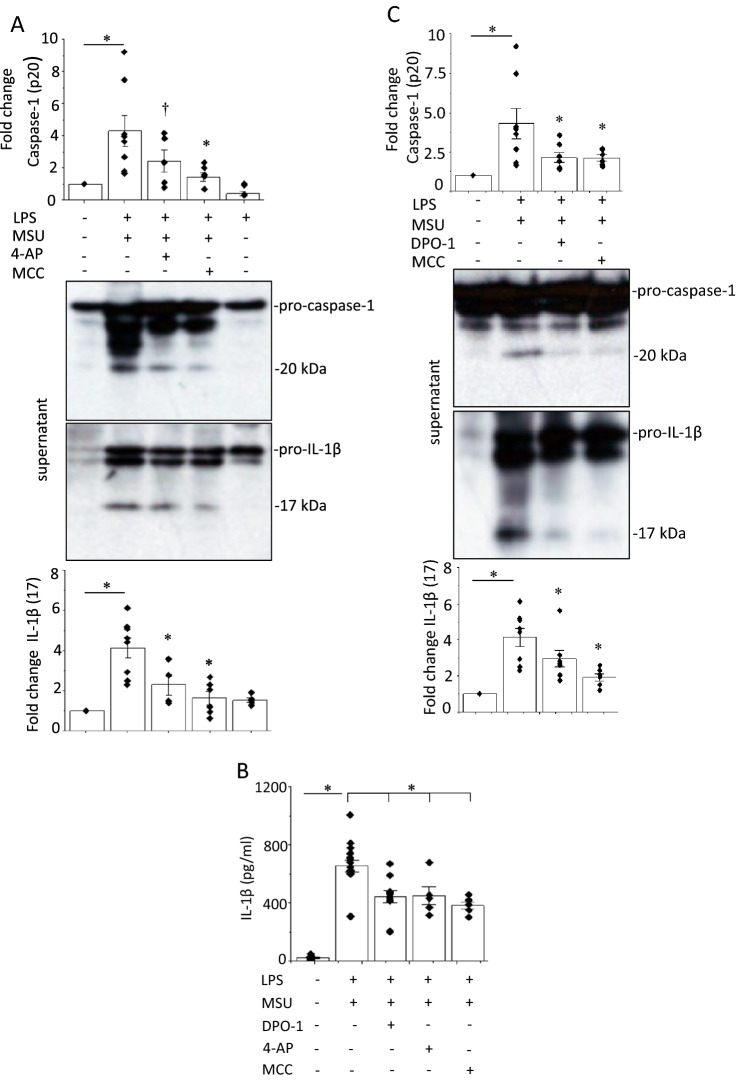
Effects of K^+^ channel blockers and a NLRP3 inflammasome inhibitor on MSU-induced activation of the NLRP3 inflammasome in J774.1 cells. The cells primed with LPS (1 μg/ml, 6 h) and subsequently stimulated with MSU (500 μg/ml, 6 h) in the presence and absence of the nonspecific K^+^ channel blocker 4-AP (6 mM), or the selective NLRP3 inflammasome inhibitor MCC950 (MCC) (50 nM) (**a**) or DPO-1 (1 μM) (**c**). The inhibitors were added 30 min before the addition of MSU. The densities of caspase-1 (p20) and IL-1β (p17) were normalized to those determined for untreated cells, as indicated by the bar graph (n = 4–8). **b** IL-1β concentrations in culture supernatants of the cells measured by ELISA (n = 5–10). *p < 0.01, ^†^*p* < 0.05 vs. LPS-primed and MSU-treated cells without administration of the inhibitors

Since K^+^ efflux is a common trigger of NLRP3 inflammasome activation [5], we first examined the effects of K^+^ channel inhibitors on the production of caspase-1 and IL-1β to determine whether K^+^ channels are responsible for NLRP3 inflammasome activation. We treated LPS-primed cells with a known NLRP3 inflammasome activator, MSU, for 6 h and measured caspase-1 and IL-1β levels using immunoblot (IB). MSU induced caspase-1 (p20) in the culture supernatants (Fig. 1a) and cell lysates (Supplementary Fig. 1), and enhanced the biologically active form of IL-1β (p17) into the supernatants (Fig. 1a). The LPS-primed cells were exposed to the non-selective voltage-gated K^+^ channel inhibitor 4-AP [6] or the selective NLRP3 inflammasome inhibitor MCC950 [3] 30 min before MSU stimulation. 4-AP and MCC950 significantly decreased the levels of both caspase-1 and IL-1 β (Fig. 1a and Supplementary Fig. 1). ELISA confirmed that MSU enhanced IL-1β secretion, which was inhibited by 4-AP and MCC950 (Fig. 1b).

**Fig. 2:**
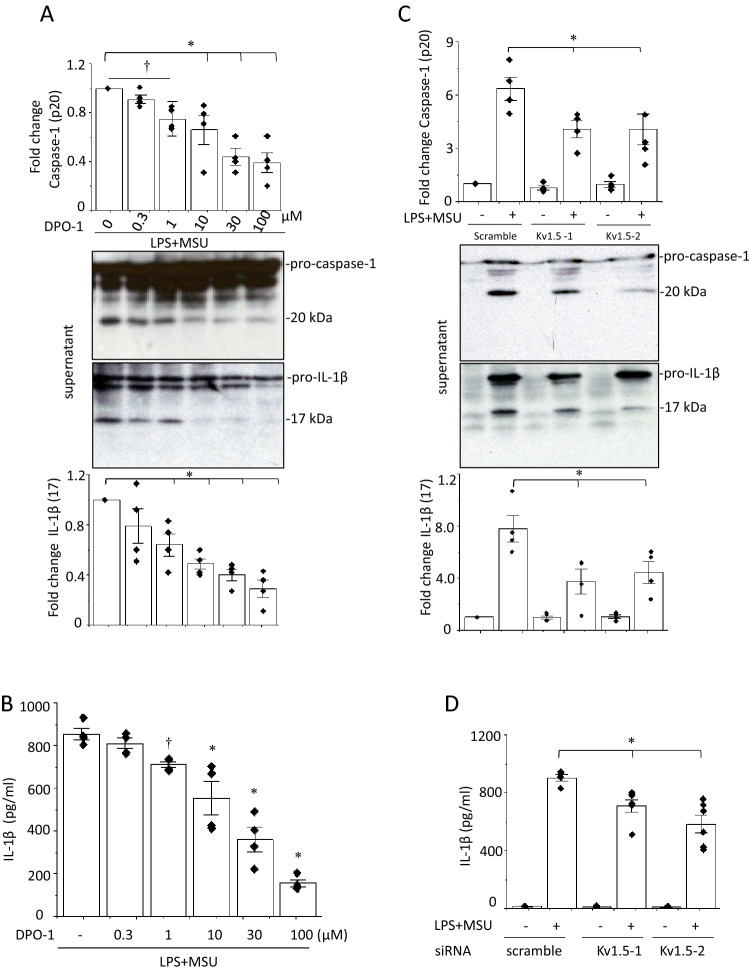
Concentration dependent effects of DPO-1 and Kv.1.5 knockdown on MSU-induced NLRP3 inflammasome activation in J774.1 cells. DPO-1 (0–100 μM) were added 30 min before the addition of MSU (**a**). 24 h after the introduction of one of two siRNAs against Kv1.5 (Kv1.5-1 and Kv1.5-2) or a scramble siRNA, LPS-primed cells were stimulated by MSU (**c**). Shown are representative blots. (n = 4). *p < 0.01, ^†^*p* < 0.05. IL-1β in the culture supernatant of the LPS-primed MSU-stimulated cells treated with DPO-1 (**b**) or transfected with one of the siRNAs against Kv1.5 or the scramble siRNA (**d**) were analyzed by ELISA (n = 4–6). *p < 0.01, ^†^p < 0.05 vs.DPO-1(−)

We attempted to identify the K^+^ channel required for activation of the NLRP3 inflammasome. Since Kv1.5 expression has been found in mouse and human macrophages, including J774.1, Raw264 and THP-1 cells [7, 13–15], we examined whether Kv1.5 channels are involved in MSU-induced NLRP3 inflammasome activation by using the selective Kv1.5 channel blocker DPO-1 [16]. DPO-1 decreased the protein levels of both caspase-1 and IL-1β (Fig. 1b, c) in a concentration-dependent manner (Fig. 2a, b). Two small interfering (si) RNAs against Kv1.5 (Kv1.5-1 and 2) decreased Kv1.5 protein levels (Supplementary Fig. 2) and suppressed MSU-induced increases in the levels of caspase-1 and IL-1β (Fig. 2c, d). The effects of DPO-1 on MSU-induced activation of the NLRP3 inflammasome in human THP-1 cells provided a similar results (Supplementary Fig. 3a, b). These data suggest that Kv1.5 is required for MSU-induced NLRP3 inflammasome activation.

Several K^+^ channels such as Kv1.3, Kir2.1 (inward-rectifier K^+^ channel), TWIK2 (two-pore domain K^+^ channel) and Kir6.2 (ATP-sensitive K^+^ channel) are expressed in J774.1 cells [12, 14, 17, 18]. To determine whether Kv1.3 regulates activation of the NLRP3 inflammasome, the selective Kv1.3 blocker PAP-1 was used [19]. PAP-1 at 2–50 nM failed to alter the levels of caspase-1 and secretion of IL-1β (Supplementary Fig. 4a–c). Two siRNAs against Kv1.3 (Kv1.3-1 and 2) both decreased the protein levels of Kv1.3 but did not reduce the expression of caspase-1 and IL-1β proteins (Supplementary Fig. 5a, b). PAP-1 effects on caspase-1 and IL-1β productions were recapitulated in human THP-1 cells (Supplementary Figs. 3b, 6). The inward-rectifier K^+^ channel inhibitor BaCl_2_ [20] was used to examine the roles of inward-rectifier K^+^ channels. BaCl_2_ at 0.1–1 mM did not change the levels of capase-1 and IL-1β (Supplementary Fig. 7a). TWIK2 and ATP-sensitive K^+^ channels mediate ATP- and glucose-induced NLRP3 inflammasome activation, respectively, in J774.1 cells [12, 17]. However, the TWIK2 channel inhibitor quinine and the ATP-sensitive K^+^ channel inhibitor glibenclamide did not alter capsase-1 and IL-1β levels in MSU-treated cells (Supplementary Fig. 7b). These results indicate that Kv1.5 regulates MSU-induced activation of the NLRP3 inflammasome in macrophages.

### Kv1.5 regulates MSU-induced activation of NLRP3 inflammasome via reductions in intracellular K^+^ levels

**Fig. 3:**
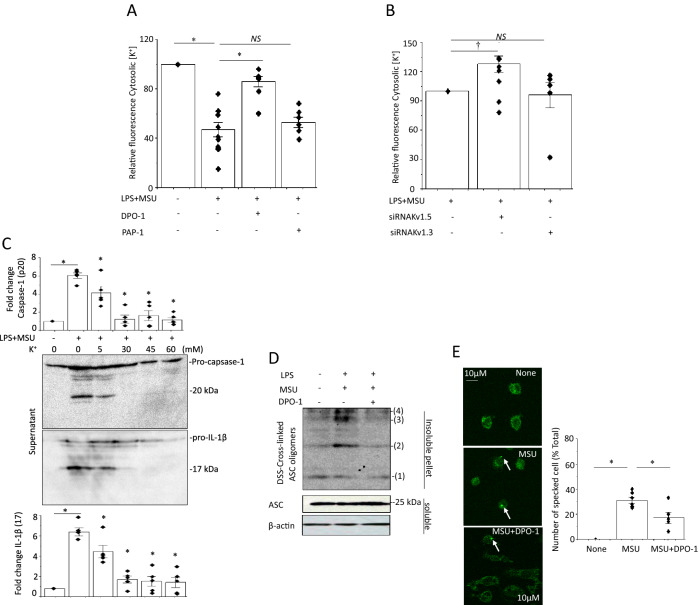
Effects of Kv1.5 on intracellular K^+^ concentration, ASC oligomerization and speck formation in J774.1 cells. Intracellular K^+^ concentrations of LPS-primed cells stimulated with MSU in either the presence or absence of DPO-1 (1 μM) or the selective Kv1.3 channel blocker PAP-1 (50 nM) (**a**), and those with the introduction of a siRNA against Kv1.5 or Kv1.3 or a scramble siRNA 24 h before the LPS and MSU treatments (**b**) (n = 5–11). *p < 0.01, †*p* < 0.05; *NS*: not statistically significant. **c** The LPS-primed and subsequently stimulated with MSU cells were incubated with OPTI-MEM containing different K^+^ concentrations (0–60 mM) overnight (n = 5). Shown are the representative blot. *p < 0.01 vs. LPS + MSU (+) & K^+^ 0 mM. **d** IBs of disuccinimidyl suberate (DSS)-crosslinked ASC oligomers in lysates from untreated cells and LPS-primed cells treated with DPO-1 (1 μM) or vehicle 30 min before MSU stimulation (n = 5). **e** Representative immunofluorescence images of ASC speck formation in LPS-primed MSU-stimulated cells with and without DPO-1 (1 μM) treatment. Arrows denote ASC specks. Averaged numbers of specked cells (% total) are shown by the bar graph (n = 5–6 fields). *p < 0.01

Whether the K^+^ efflux induced by Kv1.5 channels could trigger activation of the NLRP3 inflammasome in MSU-treated J774.1 cells, we evaluated the effects of MSU on the intracellular K^+^ concentration in the presence or absence of DPO-1. MSU caused a marked decrease in intracellular K^+^ levels (Fig. 3a). DPO-1 prevented the MSU-induced decrease in intracellular K^+^ levels. The siRNAs against Kv1.5 introduced to LPS-primed cells mimicked the effects of DPO-1 on intracellular K^+^ levels (Fig. 3b). To evaluate the role of K^+^ efflux in MSU-induced NLRP3 inflammasome activation, LPS-primed cells were incubated in OPTI-MEM with various K^+^ concentrations. MSU-induced enhancement of caspase-1 expression and IL-1β secretion was suppressed with increasing extracellular K^+^ concentrations (Fig. 3c), demonstrating that MSU-induced activation of NLRP3 inflammasome is related to K^+^ efflux and a decrease in intracellular K^+^ concentrations.

Whether Kv1.5 directly interacts with NLRP3 and/or ASC to affect NLRP3 inflammasome complex formation, we examined the association of Kv1.5 with NLRP3 and ASC by immunoprecipitation. The anti-Kv1.5 immunoprecipitates (IPs) in J774.1 cells contained neither ASC nor NLRP3. Kv1.5 was not present in either anti-ASC or anti-NLRP3 IPs (Supplementary Fig. 8a, b). Thus, Kv1.5 activates the NLRP3 inflammasome not by direct interactions with NLRP3 or ASC.j

### Kv1.5 promotes ASC oligomerization and speck formation in J774.1 cells

ASC oligomerization and speck formation are key events of the NLRP3 inflammasome activation [3, 21]. We examined the effects of Kv1.5 channel inhibition on ASC oligomerization and speck formation. When the cytosolic fraction from cell lysates was cross-linked using disuccinimidyl suberate (DSS), ASC dimers and oligomers were detected in LPS-primed and MSU-stimulated cells but not in that of untreated cells. DPO-1 inhibited ASC oligomerization (Fig. 3d). Immunofluorescence analyses showed that MSU stimulation of LPS-primed cells induced formation of ASC specks (Fig. 3e). DPO-1 treatment reduced the number of ASC specks.

### MSU enhances Kv1.5 protein expression and channel function via Hsp70

**Fig. 4:**
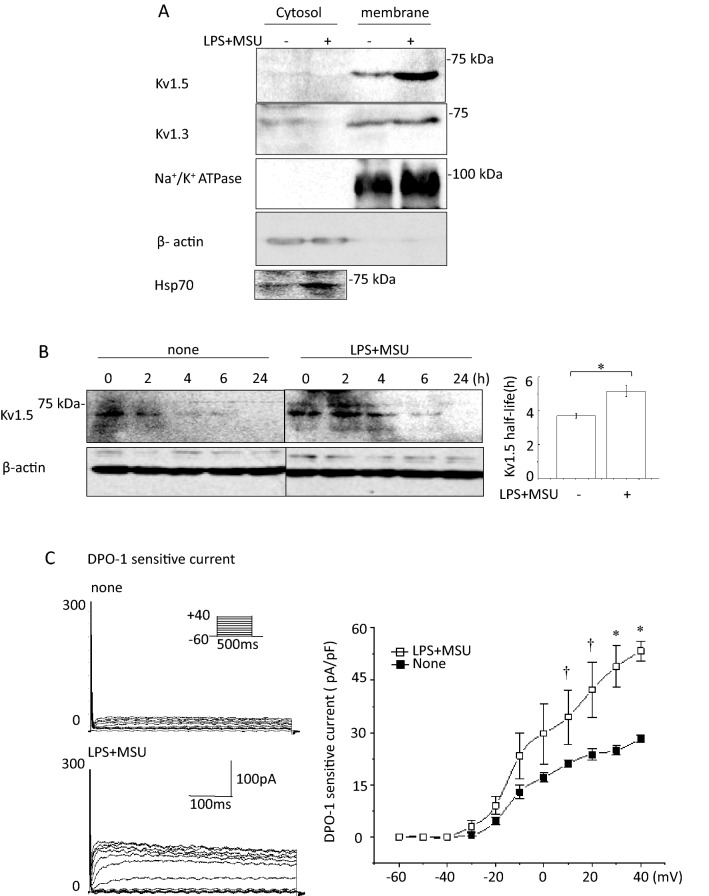
MSU effects on protein expression of K^+^ channels and Hsp70, degradation of Kv1.5 proteins and Kv1.5 channel currents in J774.1 cells. **a** IB analysis of Kv1.5 and Kv1.3 proteins and Hsp70 in the cytosolic and membrane fraction of LPS-primed and MSU-stimulated cells and untreated cells. Na^+^/K^+^ ATPase and β-actin were used as the plasma membrane and protein loading control, respectively. **b** Degradation of Kv1.5 proteins. The LPS-primed MSU-treated cells (LPS + MSU) or untreated cells (none) were chased for the indicated times after an addition of cycloheximide. Representative blots are shown. The densities of Kv1.5 was normalized to the density at time 0 and β-actin. Bar graph shows the half-life of the Kv1.5 proteins (n = 4). *p < 0.01. **c** Effects of MSU on Kv1.5 channel currents measured as DPO-1-sensitive currents. Whole-cell membrane currents were recorded from a single J774.1 cell without LPS and MSU treatments (none) and that with LPS priming and MSU stimulation (LPS + MSU). Shown are representative DPO-1-sensitive currents that were obtained by digital subtraction. Current–voltage relationships of currents are also shown (n = 6–8). *p < 0.01, ^†^*p* < 0.05 vs. None

To investigate how MSU enhanced Kv1.5 expression, we examined the effects of MSU on Kv1.5 protein localization and levels in LPS-primed J774.1 cells using fractionation experiment. The Kv1.5 levels on the plasma membrane, not in the cytosol, and Hsp70 expression were increased by MSU (Fig. 4a). The half-life of Kv1.5 proteins was 3.7 ± 0.1 h in untreated cells and was prolonged to 5.1 ± 0.3 h in MSU-treated cells (Fig. 4b). Kv1.5 channel currents in J774.1 cells were recorded as DPO-1-sensitive currents using the patch clamp technique. In cells without LPS and MSU treatment (none), depolarization pulses activated time-dependent outward currents, which were completely abolished by DPO-1. LPS priming followed by MSU treatment augmented DPO-1 sensitive currents (Fig. 4c).

**Fig. 5:**
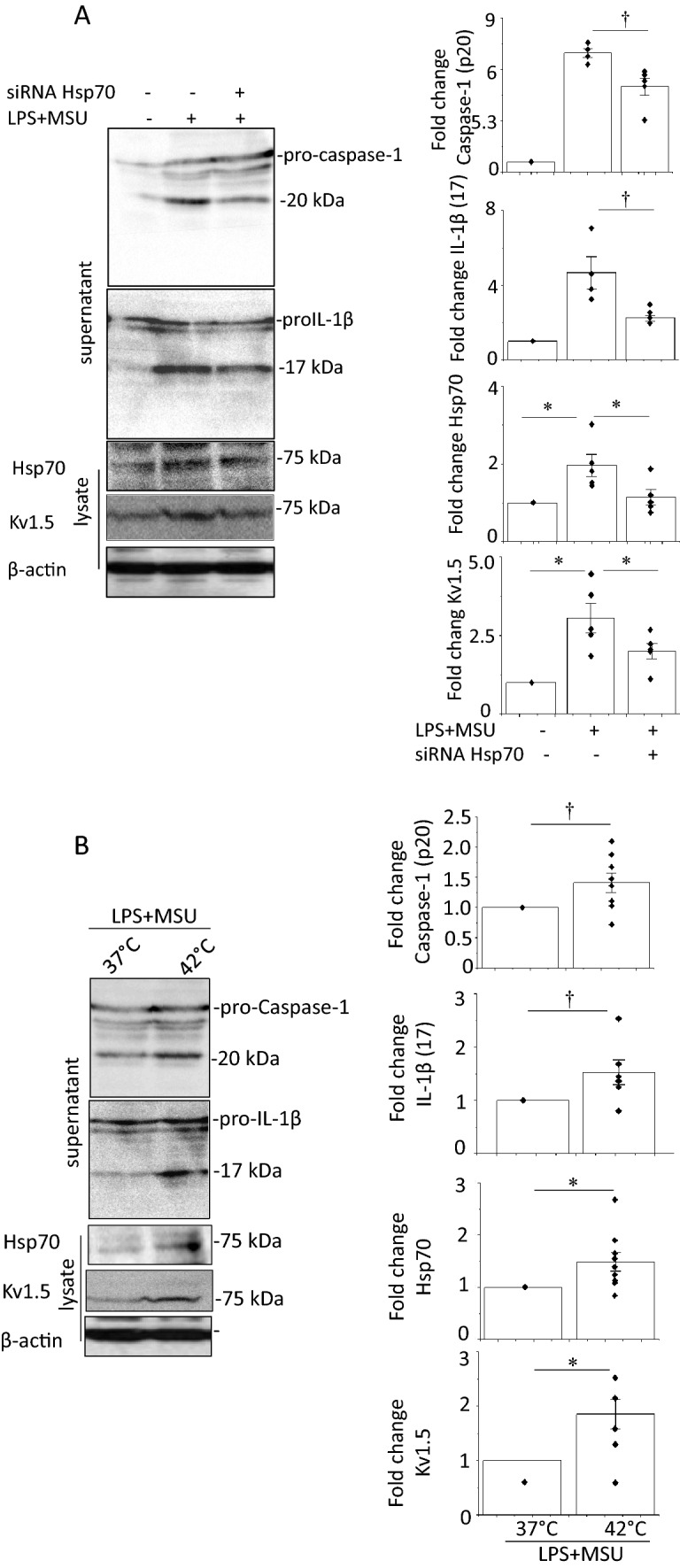
Effects of a siRNA against Hsp70 and heat shock (HS) on MSU-induced NLRP3 inflammasome activation and the expression of Kv1.5 protein. The LPS-primed J774.1 cells treated with MSU 24 h after the introduction of either a scramble siRNA or a siRNA against Hsp70 (**a**) (n = 4–5) or pre-treated at 42 °C for 1 h (**b**) (n = 5–9). Representative blots are shown. Image densities were normalized to those in the MSU-untreated cells transfected with a scramble siRNA or non-HS control cells. ^†^*p* < 0.05, *p < 0.01

We examined whether Hsp70 is involved in the MSU-induced enhancement of Kv1.5 expression. Anti-Kv1.5 IPs in J774.1 cells contained Hsp70; furthermore, anti-Hsp70 IPs contained Kv1.5 (Supplementary Fig. 8c). To study the roles of Hsp70 in MSU-induced NLRP3-inflammasome activation, an siRNAs against Hsp70 was introduced into LPS-primed and MSU-treated cells. The siRNA inhibited MSU-induced increases in caspase-1, IL-1β, and Kv1.5, as well as Hsp70 (Fig. 5a). Hsp70 in J774.1 cells was increased by heat shock (HS) treatment at 42 °C for 1 h; HS enhanced the MSU-induced increases in caspase-1, IL-1β, and Kv1.5 levels (Fig. 5b). These results suggest that MSU-induced enhancement of Kv1.5 protein expression and resulting activation of the NLRP3-inflammasome could be attributed to post-transcriptional modification of Kv1.5 proteins by Hsp70.

### Effects of CM from MSU-activated macrophages on NLRP3 inflammasome activity in HL-1 mouse atrial myocytes

**Fig. 6:**
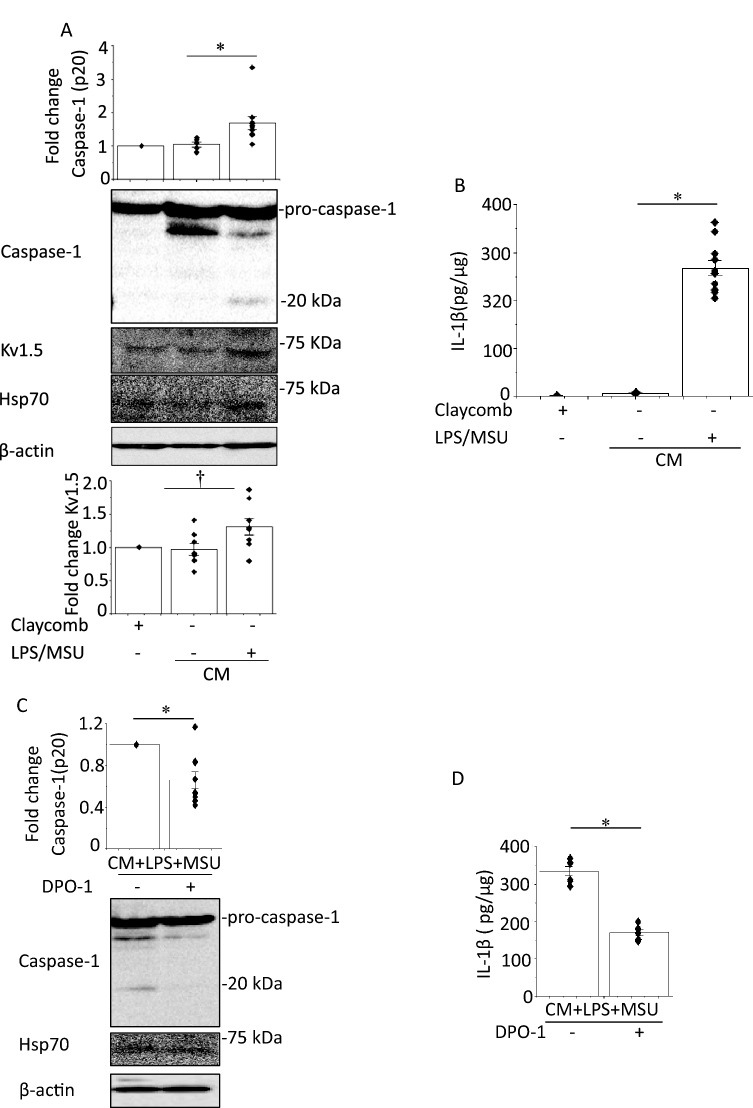

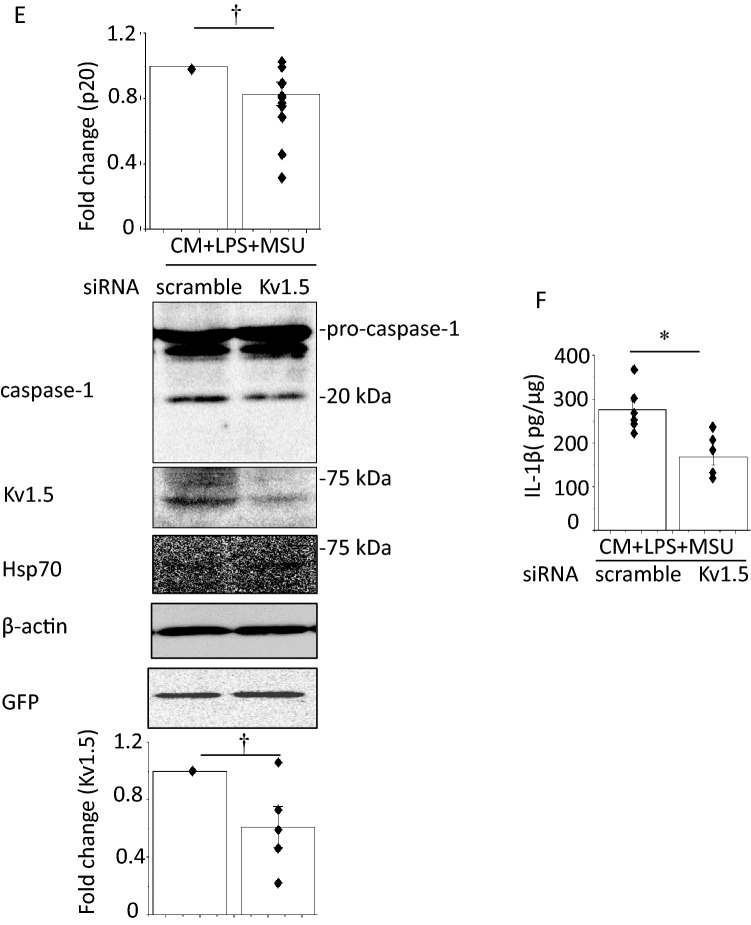
Effects of the CM from LPS-primed and MSU-stimulated macrophages on activation of NLRP3 inflammasome and Kv1.5 in HL-1 atrial myocytes. HL-1 cells were incubated with Claycomb, the CM from J774.1 cells treated with vehicle (LPS/MSU−) or the CM from LPS-primed MSU-treated cells (LPS/MSU+) overnight. Shown are representative IBs (**a**) (n = 5–8). **b** IL-1β concentrations in the cell lysates measured by ELISA (n = 4–11). HL-1 cells were treated with DPO-1 (1 μM) or a vehicle 30 min before the incubation with CM + LPDS + MSU overnight. Show are representative blots (**c**) (n = 9) and IL-1β concentrations in the cell lysates measured by ELISA (**d**) (n = 6). *p < 0.01. HL-1 cells were transfected with a scramble siRNA or a siRNA against Kv1.5 24 h before incubation with CM + LPS + MSU. Show are representative blots (**e**) (n = 5–11) and IL-1β concentrations measured by ELISA (**f**) (n = 6). ^†^*p* < 0.05, *p < 0.01

**Fig. 7:**
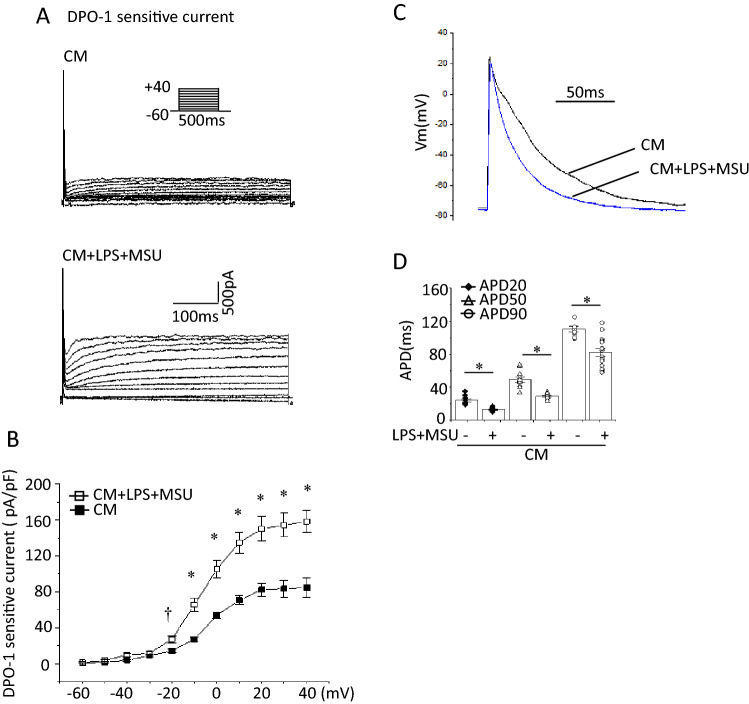
Effects of the CM + LPS + MSU on DPO-1-sensitive currents and APD in HL-1 cells. **a** Whole-cell membrane currents recorded from a single HL-1 cell before and after application of DPO-1(1 μM). DPO-1-seseitive currents were obtained by digital subtraction. Current recordings were performed after incubation with the untreated control CM (CM) or CM + LPS + MSU overnight. **b** Current–voltage relationships of the peak DPO-1-sensitive current (n = 9–15). ^†^*p* < 0.05, *p < 0.01 vs. the control CM. **c** Action potentials recorded after incubation with the control CM or CM + LPS + MSU overnight. **d** APD_20_, APD_50_ and APD_90_ values summarized as a bar graph (n = 8–15). *p < 0.01

Enhanced cardiomyocyte NLRP3 inflammasome signaling promotes AF with increased *I*_Kur_ [4]. To assess the direct effects of MSU on NLPR3 inflammasome in atrial myocytes, we stimulated LPS-treated HL-1 cells with MSU for 6 h. Caspase-1 or IL-1β bands could not be detected in supernatants or cell lysates (Supplementary Fig. 9). Therefore, we hypothesized that MSU activates NLRP3 inflammasome in HL-1 cells via stimulating macrophages. HL-1 cells were incubated with Claycomb medium, the CM from untreated J774.1 cells (control CM) or the CM obtained from LPS-primed and MSU-treated J774.1 cells (CM + LPS + MSU) overnight. The bands of caspase-1 in cell lysates were undetectable in the HL-1 cells cultured with either Claycomb medium or the control CM. Exposure to CM + LPS + MSU induced caspase-1 bands and increased IL-1β expression measured by ELISA as well as enhanced Kv1.5 and Hsp70 expression (Fig. 6a, b). DPO-1 (1 μM) was added to the medium 30 min prior to exposure to CM + LPS + MSU or an siRNA against Kv1.5 was introduced into HL-1 cells 24 h before exposure to CM + LPS + MSU. Both DPO-1 and knockdown of Kv1.5 decreased the levels of caspase-1 and IL-1β in the HL-1 cells exposed to CM + LPS + MSU without alteration of Hsp70 expression (Fig. 6c–f). Depolarizing test pulses activated time-dependent outward currents in HL-1 cells exposed to the control CM, which were completely abolished by DPO-1 (Fig. 7a, b). HL-1 cells exposed to CM + LPS + MSU showed larger DPO-1 sensitive currents and shorter APDs than those exposed to the control CM without differences in resting membrane potentials (Fig. 7b–d). These findings suggest that as a paracrine action the CM from MSU-activated macrophages could induce activation of NLRP3 inflammasome and enhancement of Kv1.5 channel currents to shorten APD in HL-1 cells.

## Discussion

MSU-induced gout flares are characterized by activation of the NLRP3 inflammasome which requires K^+^ efflux [5]. Mouse bone marrow-derived macrophages (BMDMs) were incubated in K^+^-free medium without any stimuli inducing IL-1β secretion, indicating that K^+^ efflux mediates activation of the NLRP3 inflammasome [5]. Kv1.5 and other K^+^ channels are expressed in macrophages [12–15, 17, 18] and involved in immune responses [22, 23]. K^+^ efflux through Kv1.5 channels forms outward *I*_Kur_ that regulates APD [4, 6]. In the current study, MSU-triggered decline in the intracellular K^+^ concentration accompanied enhanced caspase-1 formation and IL-1β release. Blocking Kv1.5 by the selective inhibitor DPO-1 decreased outward K^+^ currents corresponding to *I*_Kur_ and attenuated MSU-induced production of caspase-1 and IL-1β (Fig. 1b, c). The role of Kv1.5 in MSU-induced activation of NLRP3 inflammsome was confirmed by siRNA knockdown of Kv1.5 and was recapitulated in human macrophages. Kv1.5 protein expression is closely related to *I*_Kur_ and intracellular K^+^ concentrations in macrophages (Figs. 3b, 4a and c). DPO-1 and siRNAs against Kv1.5 similarly and effectively prevented all of them (Figs. 1c, 2c), even though DPO-1 did not significantly change Kv1.5 protein expression (Supplementary Fig. 10). Furthermore, extracellular solutions of higher K^+^ concentrations (30–60 mM) also clearly abolished MSU-induced NLRP3 inflammsome activation (Fig. 3c). Taken together, Kv1.5 mediated K^+^ efflux that decreases intracellular K^+^ concentrations is responsible for the MSU-induced activation of the NLRP3 inflammasome.

Molecular chaperones participate in the biogenesis of K^+^ channels, such as Kv1.5 and hERG. Hsp70 stabilizes these channel proteins and enhances K^+^ currents by post-transcriptional modifications [24, 25]. The direct interaction of Hsp70 and Kv1.5 proteins has been confirmed (Supplementary Fig. 8c). HS and cell stress induce Heat shock proteins (Hsps), including Hsp70. Hsp70 expressions are increased by soluble uric acid and IL-1β in HL-1 and Hela cells, respectively [8, 26]. MSU-treated macrophages contained more than 30 times higher concentrations of IL-1β than that in untreated macrophages (Fig. 1b). IL-1β produced by activated NLRP3 inflammasome may further enhance Hsp70 expression which exaggerates NLRP3 inflammsome activation by increasing Kv1.5 expression.

Enhanced cardiomyocyte NLPR3 inflammasome activity promotes AF [4]. MSU directly activates NLRP3 inflammasome in macrophages [2]. MSU did not directly induce capsase-1 expression and IL-1β secretion in HL-1 cells (Supplementary Fig. 9), whereas the CM from macrophages treated with LPS and MSU increased capsase-1 expression, IL-1β secretion, Kv1.5 protein expression and Kv1.5 channel currents, and shortened APD in HL-1 cells (Figs. 6a, b, 7a–d). These results suggest that activation of NLRP3 inflammasome and electrical remodeling in HL-1 cells are induced by MSU-stimulated macrophages. In vivo conditions cardiomyocytes contact with resident and infiltrated macrophages. Increased infiltration of macrophages and elevated IL-1β production have been found in the atrial tissue of AF patients [27]. Macrophages are polarized by LPS to be differentiated into pro-inflammatory type [27]. Administration of LPS for two weeks increased pro-inflammatory macrophage in the atrium and AF incidence with shortened atrial effective refractory period in both canine and mouse AF models [27]. Thus, pro-inflammatory macrophages play a key role in the occurrence of NLRP3 inflammasome-related AF. The resident macrophages in various tissues exert distinct properties from monocyte-derived macrophages. In general monocyte-derived macrophages, such as J774.1 cells, have an inflammatory phenotype by production of IL-1β, whereas the tissue resident macrophages, such as cardiac resident macrophages, possess an anti-inflammatory phenotype [28, 29]. We assume that the CM from LPS-primed and MSU-treated inflammatory phenotype macrophages mimic the effect of gout-activation of NLRP3 inflammsome in macrophages on cardiomyocytes in vitro.

The active NLRP3 promotes automaticity of atrial myocytes and shortens atrial refractory period by enhancing RYR2-mediated Ca^2+^ release and Kv1.5-mediated *I*_Kur_ [4]_._ In a mouse model, high fat diet induced activation of the NLRP3 inflammasome and upregulation of Kv1.5 protein expression in atrial tissue with the abbreviation of atrial-effective refractory period [30]. CM + LPS + MSU enhanced- Kv1.5 expression and channel currents, which activate NLRP3 inflammasome with shortening of APD in HL-1 cells (Figs. 6, 7). These alterations of electrical properties are involved in the development of AF and suggest that MSU-induced activation of the NLRP3 inflammasome in macrophages upregulates Kv1.5 expression in HL-1 cells, which plays a major role in APD shortening and the development of AF in atrial myocytes under hyperuricemia.

Several limitations of the present study should be addressed. First, we mainly used mouse macrophage and atrial myocyte cell lines. Whether our conclusions are also applicable to human primary macrophages and cardiomyocytes and gout in vivo models is needed to be further defined in future studies. Second, the exact factors in the CM + LPS + MSU responsible for activation of NLRP3 inflammasome in cardiomyocytes remain to be elucidated. Acute application of IL-1β causes NLRP3 inflammasome activation in HL-1 cells [31]. IL-1β probably exerts a major contribution to activation of NLRP3 inflammasome and enhancement of Kv1.5 expression in cardiomyocytes. However, the CM + LPS + MSU may contain unknown multiple biological mediators other than IL-1β. Rather than using recombinant or synthetic cytokines, the use of CM + LPS + MSU may evoke more biologically relevant responses. Third, several K^+^ channels are expressed on the HL-1 cell membrane. The roles of other K^+^ channels should be examined in more detail. Fourth, how K^+^ efflux leads to activation of the NLRP3 inflammasome remains unknown.

In summary, MSU activates the NLRP3 inflammasome in macrophages via enhancement of Kv1.5 mediated K^+^ efflux. MSU-stimulated macrophages activate the NLRP3 inflammasome and promote electrical remodeling and inflammation in atrial myocytes. Kv1.5 may be a potential therapeutic target for gout-related inflammation and AF.

## Supplementary Information

Below is the link to the electronic supplementary material.

Supplementary file1 (DOCX 31 kb)

Supplementary file2 (PPTX 7013 kb)

## Data Availability

The datasets analyzed in the present study are available from the corresponding author on reasonable request.
