# Supplementary material for: Kv1.5 channel mediates monosodium urate-induced activation of NLRP3 inflammasome in macrophages and arrhythmogenic effects of urate on cardiomyocytes
Source: Mol Biol Rep. 2022 Apr 4;49(7):5939–52. doi: 10.1007/s11033-022-07378-1 (PMC9270276; doi:10.1007/s11033-022-07378-1)
Supplement: Supplementary file 1 — Supplementary file1 (DOCX 31 kb) [file 11033_2022_7378_MOESM1_ESM.docx]

# Supplementary Figure Legends

Fig. S1

Effects of K^+^ channels inhibitors on caspsase-1 level in J774.1 cell lysates.

Representative immunoblot (IB) analysis of caspase-1 in LPS-primed cells stimulated with MSU (500μg/ml, 6 h) in the presence and absence of 4-AP, MCC950 (MCC), or DPO-1. The inhibitors were added 30min before the addition of MSU (n=3).

Fig. S2

Kv1.5 channel expression in J774.1 cells.

The cell lysates from J774.1 cells were subjected to IB with a Kv1.5 antibody 24 h after introduction of a siRNAs against Kv1.5 (Kv1.5-1, Kv1.5-2) or a scramble siRNA.

Fig. S3

Effects of Kv1.5 and Kv1.3 channel blockers on MSU-induced activation of the NLRP3 inflammasome in the human macrophage THP-1.

PMA-differentiated THP-1 cells were primed with LPS (1μg/ml) for 6 h, and DPO-1 (1μM), PAP-1 (50nM), or MCC (50nM) were added to the cells 30 min before stimulation with MSU (500μg/ml) for 6 h.

(**A**) Caspase-1 and IL-1β were subjected to IB. The densities of caspase-1 and IL-1β were normalized to those determined for untreated cells (n= 5-7). **p*<0.01, †p<0.05

(**B**) IL-1β concentrations in the supernatants were measured by ELISA (n= 4-10). **p*< 0.01, † p<0.05.

*NS*: not statistically significant.

Fig. S4

Effects of a selective Kv1.3 blocker PAP-1 on MSU-induced NLRP3 inflammasome activation in J774.1 cells.

The LPS-primed cells were stimulated with MSU in the presence and absence of PAP-1 (50nM) (**A**) or PAP-1 at different concentrations (**B**). PAP-1 was added 30min before the addition of MSU. Shown are representative IB with the indicated antibodies (n=4-8).

(**C**) IL-1β concentrations in the supernatants were measured by ELISA (n=5).

*NS*: not statistically significant.

Fig. S5

Effects of Kv1.3 knockdown on expression of Kv1.3 protein and MSU-induced NLRP3 inflammasome activation in J774.1 cells.

(**A**) The cells were introduced with a scramble siRNA or a siRNA against Kv1.3 (Kv1.3-1, Kv1.3-2). Shown are representative IB with the indicated antibodies.

(**B**) 24 h after the introduction of a pair of siRNAs against Kv1.3 or a scramble siRNA, LPS-primed cells were stimulated by MSU. Shown are representative IB. The densities of caspase-1 and IL-1β were normalized to those determined for the cells transfected with the scramble siRNA without LPS or MSU treatment (n=4).

*NS*: not statistically significant.

Fig. S6

Effects of the Kv1.3 inhibitor on MSU-induced NLRP3 inflammasome activation in the human THP-1 cells.

PMA-differentiated THP-1 cells were primed with LPS (1μg/ml) for 6 h and then stimulated with MSU (500μg/ml) for 6h in the presence and absence of PAP-1 (50nM). PAP-1 was added 30min before the addition of MSU. Shown are representative IB (n= 4–7).

*NS*: not statistically significant.

Fig. S7

Effects of Kir2.1, TWIK2 and K_ATP_ channel inhibitors on MSU-induced NLRP3 inflammasome activation in J774.1 cells.

(**A**) Effects of Kir2.1 channel inhibitor on MSU-induced NLRP3 inflammasome activation. The LPS-primed cells were stimulated with MSU for 6 h in presence and absence of BaCL_2_ (0.1-1mM). BaCL_2_ was added 30min before the addition of MSU. Shown are representative IB. The densities of caspase-1 and IL-1β were normalized to those determined for vehicle treated cells (n=5).

(**B**) Effects of TWIK2 and K_ATP_ channel inhibitors on MSU-induced NLRP3 inflammasome activation. The LPS-primed cells were stimulated with MSU in presence and absence of K_ATP_ inhibitor glibenclamide or the TWIK2 inhibitor quinine. The channel inhibitors were added 30min before the addition of MSU. Shown are representative IB. The densities of capsase-1 and IL-1β were normalized to those determined for vehicle treated cells (n=4).

Fig. S8

Association of Kv1.5 proteins with ASC, NLRP3 and Hsp70 proteins.

(**A**) Anti-Kv1.5 and anti-ASC IPs from J774.1 cells were subjected to IB with anti-ASC and Kv1.5 antibodies, respectively. “No 1^st^” ab represents a negative control with no primary antibody added and “input” is the positive control.

(**B**) Anti-Kv1.5 and anti-NLRP3 IPs from J774.1 cells were subjected to IB with anti-NLRP3 and Kv1.5 antibodies, respectively.

(**C**) Anti-Kv1.5 and anti-Hsp70 IPs from J774.1 cells were subjected to IB with anti-Hsp70 and anti-Kv1.5, respectively.

Fig. S9

Effects of MSU on activation of the NLRP3 inflammasome in HL-1 cells.

Representative IB analysis of caspase-1 and IL-1β from supernatant or cell lysate of HL-1 cells primed with LPS (1μg/ml, 6 h) and subsequently stimulated with MSU (500μg/ml, 6 h). n=3.

Fig. S10

Effects of DPO-1 on Kv1.5 protein expressions in J774.1 cells.

The LPS-primed cells were stimulated with MSU for 6 h in presence and absence of DPO-1 (1μM). DPO-1 was added 30min before the addition of MSU. The cell lysates were subjected to IB with the indicated antibodies. Shown are representative IB. n=4.
