# Supplementary material for: Kv1.5 channel mediates monosodium urate-induced activation of NLRP3 inflammasome in macrophages and arrhythmogenic effects of urate on cardiomyocytes
Source: Mol Biol Rep. 2022 Apr 4;49(7):5939–52. doi: 10.1007/s11033-022-07378-1 (PMC9270276; doi:10.1007/s11033-022-07378-1)
Supplement: Supplementary file 2 — Supplementary file2 (PPTX 7013 kb) [file 11033_2022_7378_MOESM2_ESM.pptx]

## Slide 1
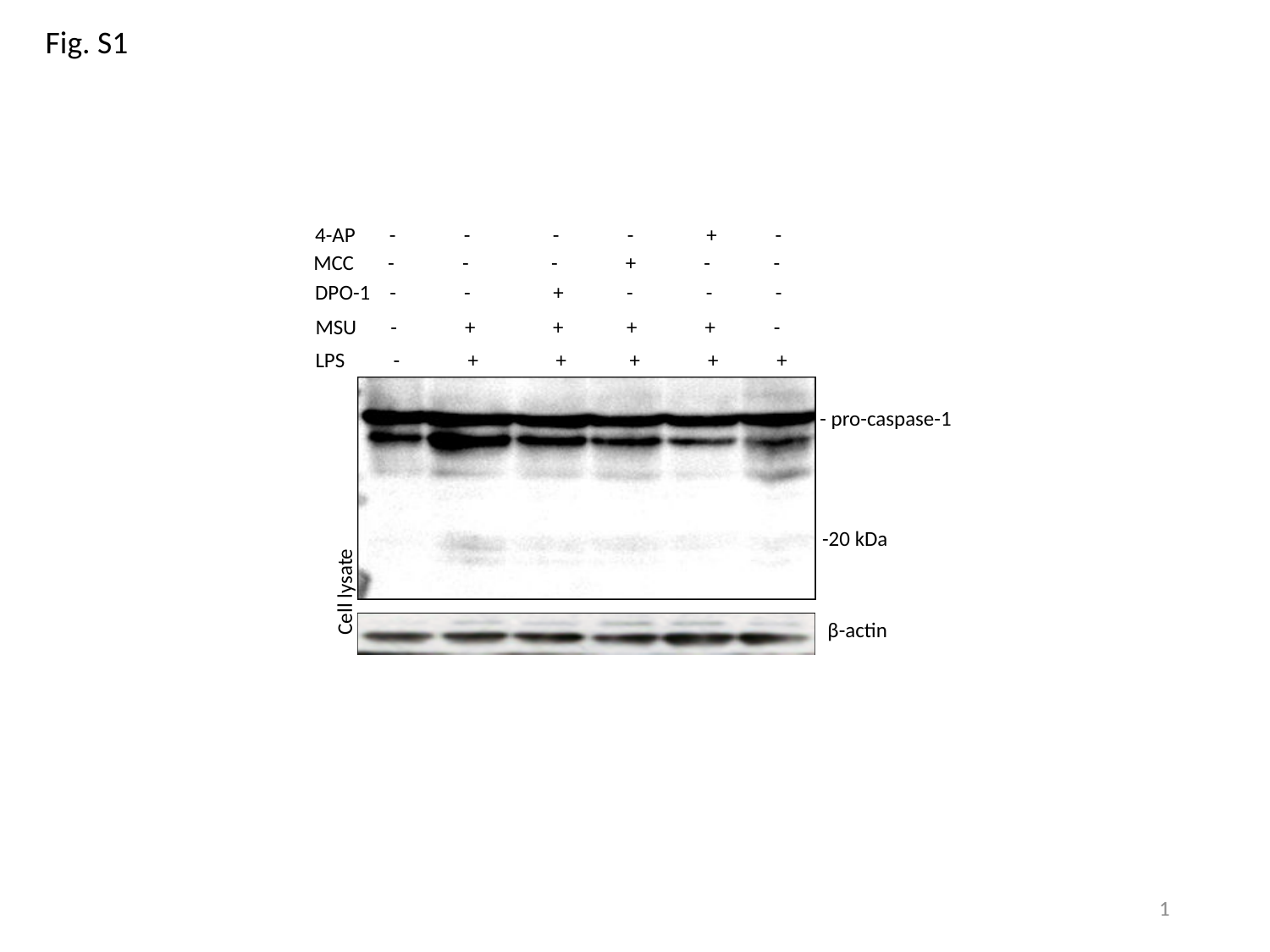

Fig. S1
4-AP - - - - + -
DPO-1 - - + - - -
MSU - + + + + -
LPS - + + + + +
- pro-caspase-1
-20 kDa
Cell lysate
β-actin
MCC - - - + - -
1

## Slide 2
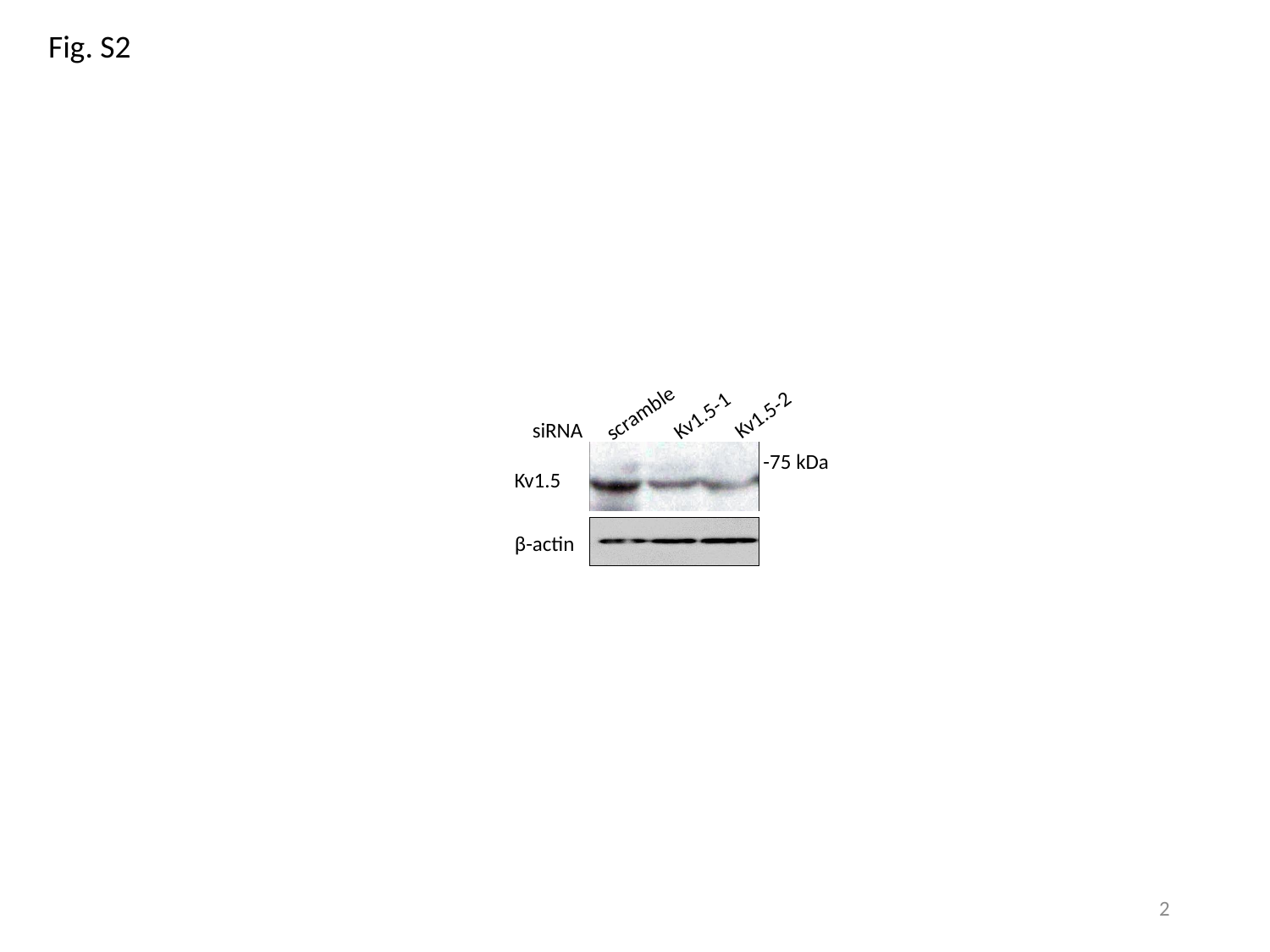

Fig. S2
scramble
Kv1.5-2
Kv1.5-1
-75 kDa
Kv1.5
siRNA
β-actin
2

## Slide 3
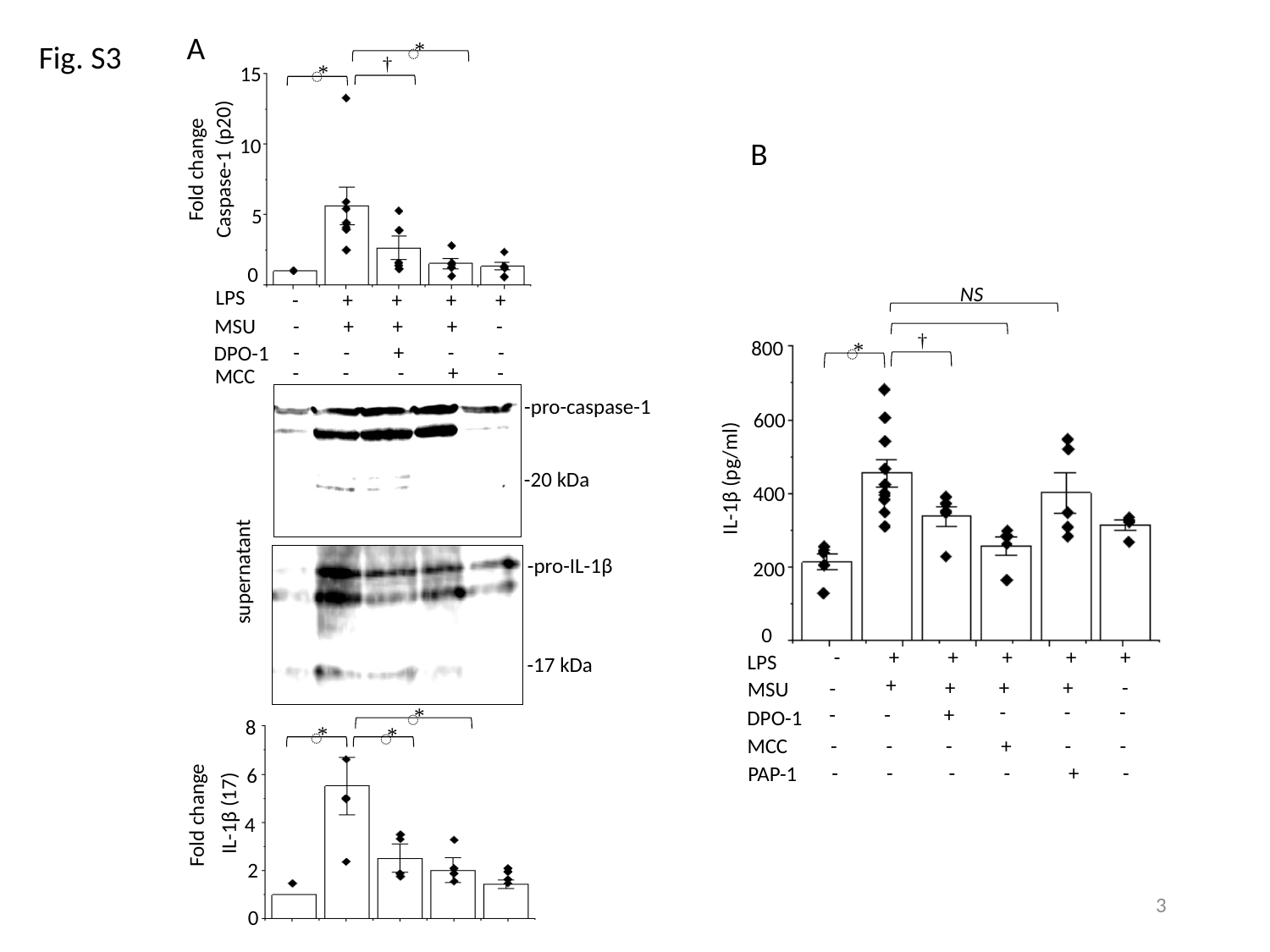

A
Fig. S3
⃰
†
15
⃰
10
5
0
 Fold change
 Caspase-1 (p20)
B
NS
†
800
⃰
600
IL-1β (pg/ml)
400
200
0
 - + + + + +
LPS
 -
+
+ + + -
MSU
 - - +
- - -
DPO-1
 -
- - + - -
MCC
 - -
- - + -
PAP-1
LPS
- + + + +
- + + + -
MSU
- - + - -
DPO-1
- - - + -
MCC
-pro-caspase-1
-20 kDa
supernatant
-pro-IL-1β
-17 kDa
⃰
8
⃰
⃰
6
Fold change
 IL-1β (17)
4
2
0
3

## Slide 4
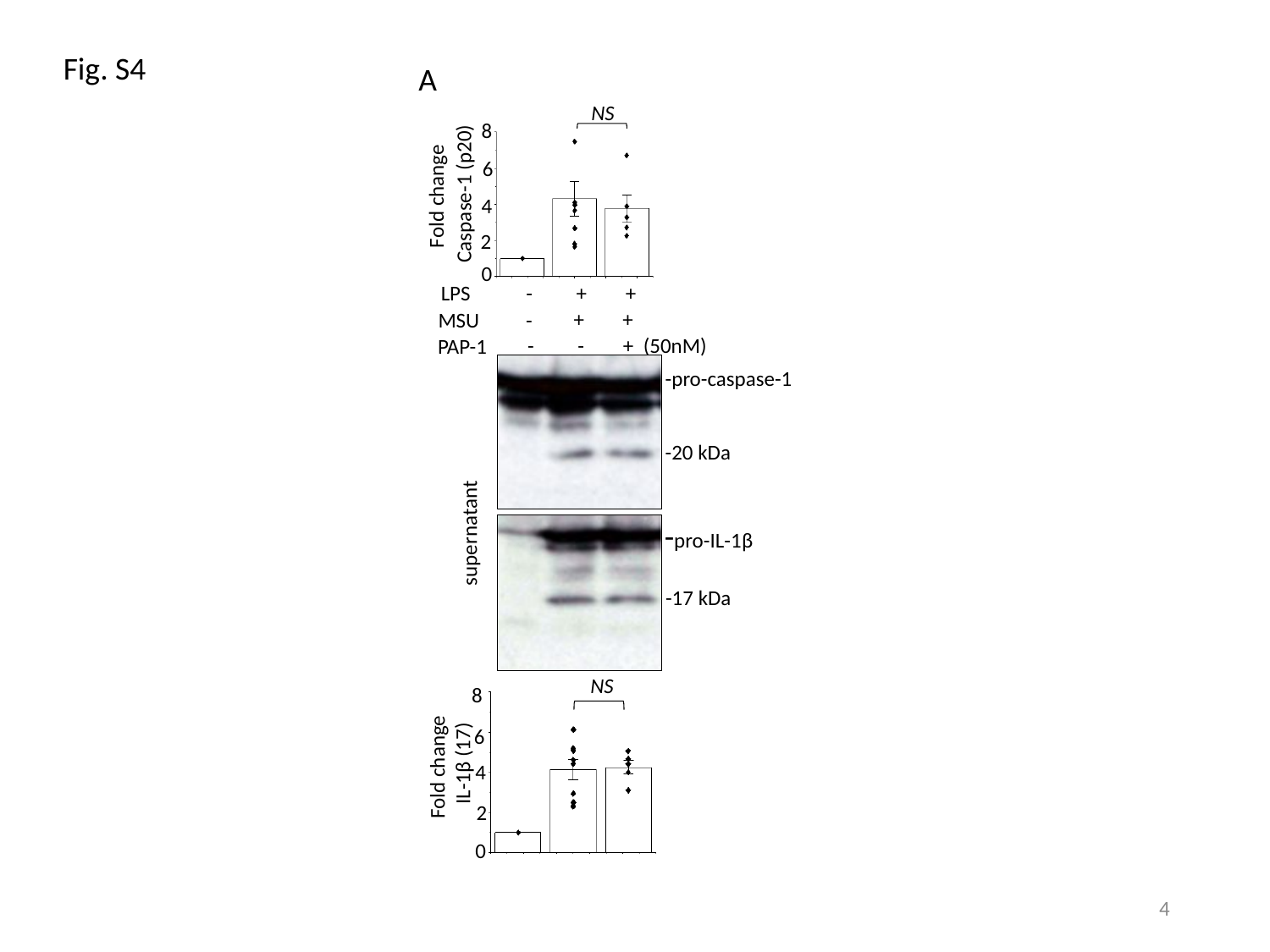

Fig. S4
A
NS
8
6
 Fold change
Caspase-1 (p20)
4
2
0
LPS
- + +
MSU
-
- - + (50nM)
PAP-1
+
+
-pro-caspase-1
-20 kDa
-pro-IL-1β
supernatant
-17 kDa
NS
8
6
Fold change
 IL-1β (17)
4
2
0
4

## Slide 5
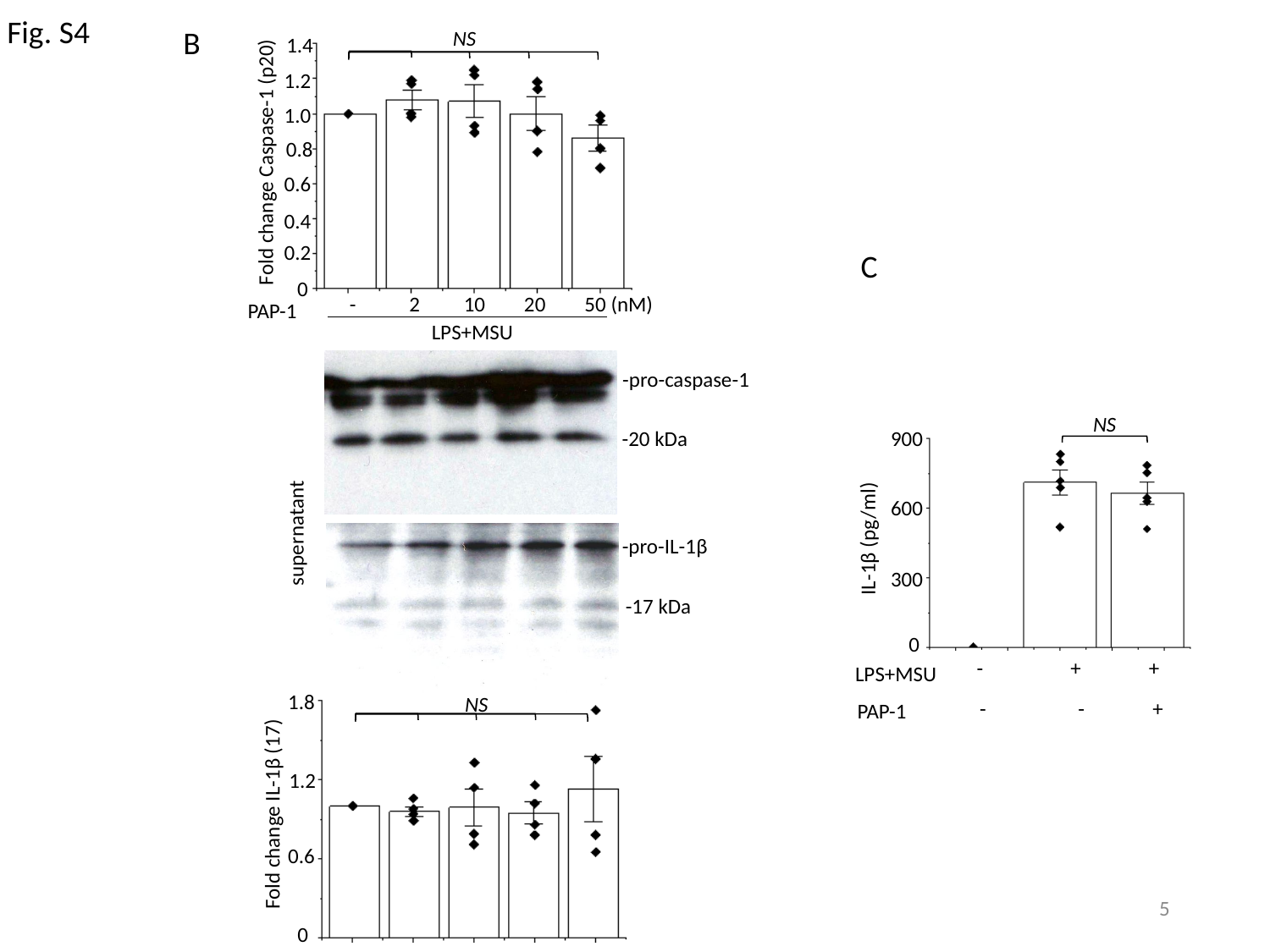

Fig. S4
B
NS
1.4
1.2
1.0
0.8
0.6
0.4
0.2
0
- 2 10 20 50 (nM)
PAP-1
LPS+MSU
-pro-caspase-1
-20 kDa
supernatant
-pro-IL-1β
-17 kDa
1.8
NS
1.2
0.6
0
 Fold change Caspase-1 (p20)
C
NS
900
IL-1β (pg/ml)
600
300
0
LPS+MSU
- + +
 - - +
PAP-1
 Fold change IL-1β (17)
5

## Slide 6
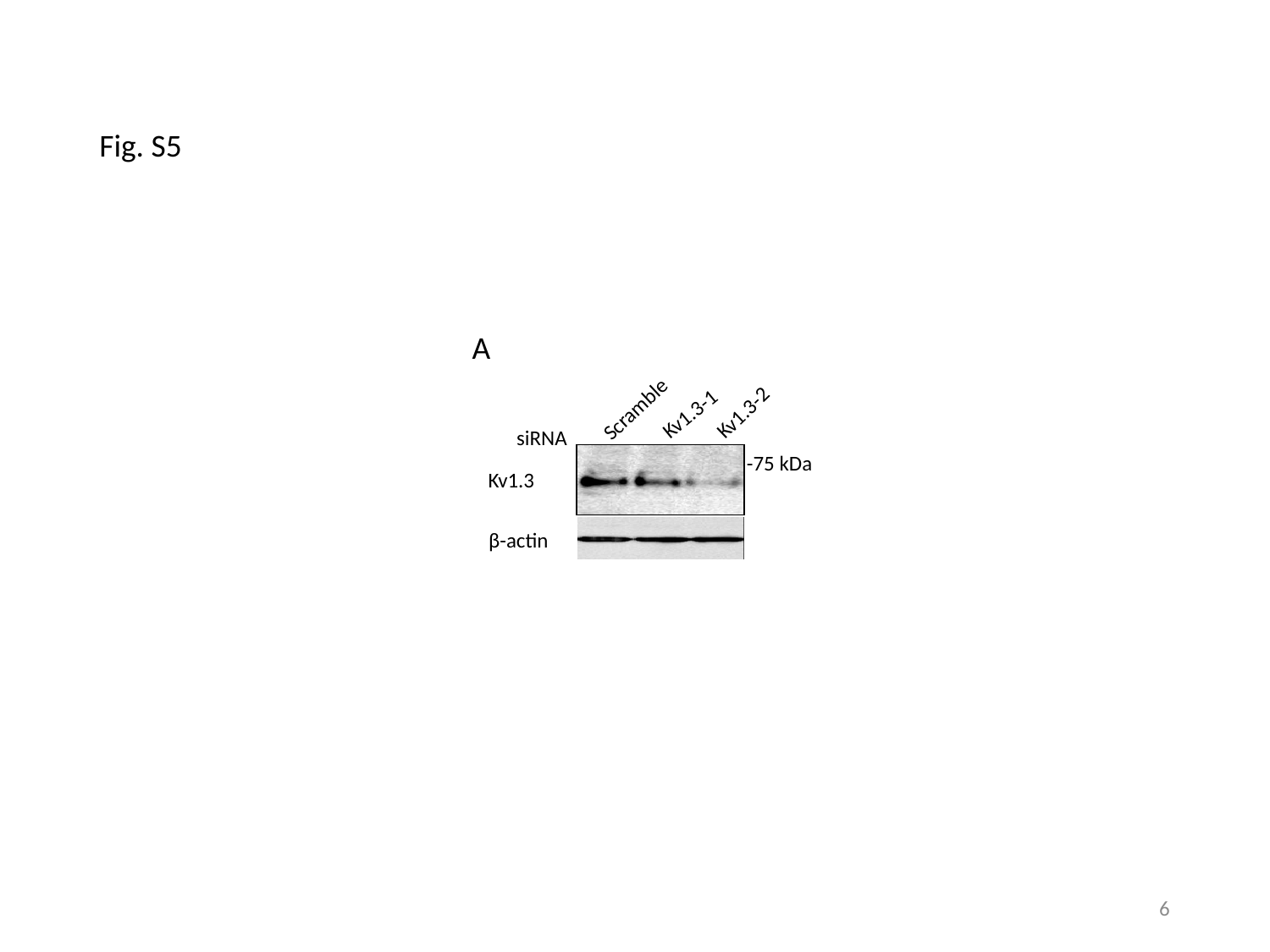

Fig. S5
A
Scramble
Kv1.3-2
Kv1.3-1
-75 kDa
Kv1.3
siRNA
β-actin
6

## Slide 7
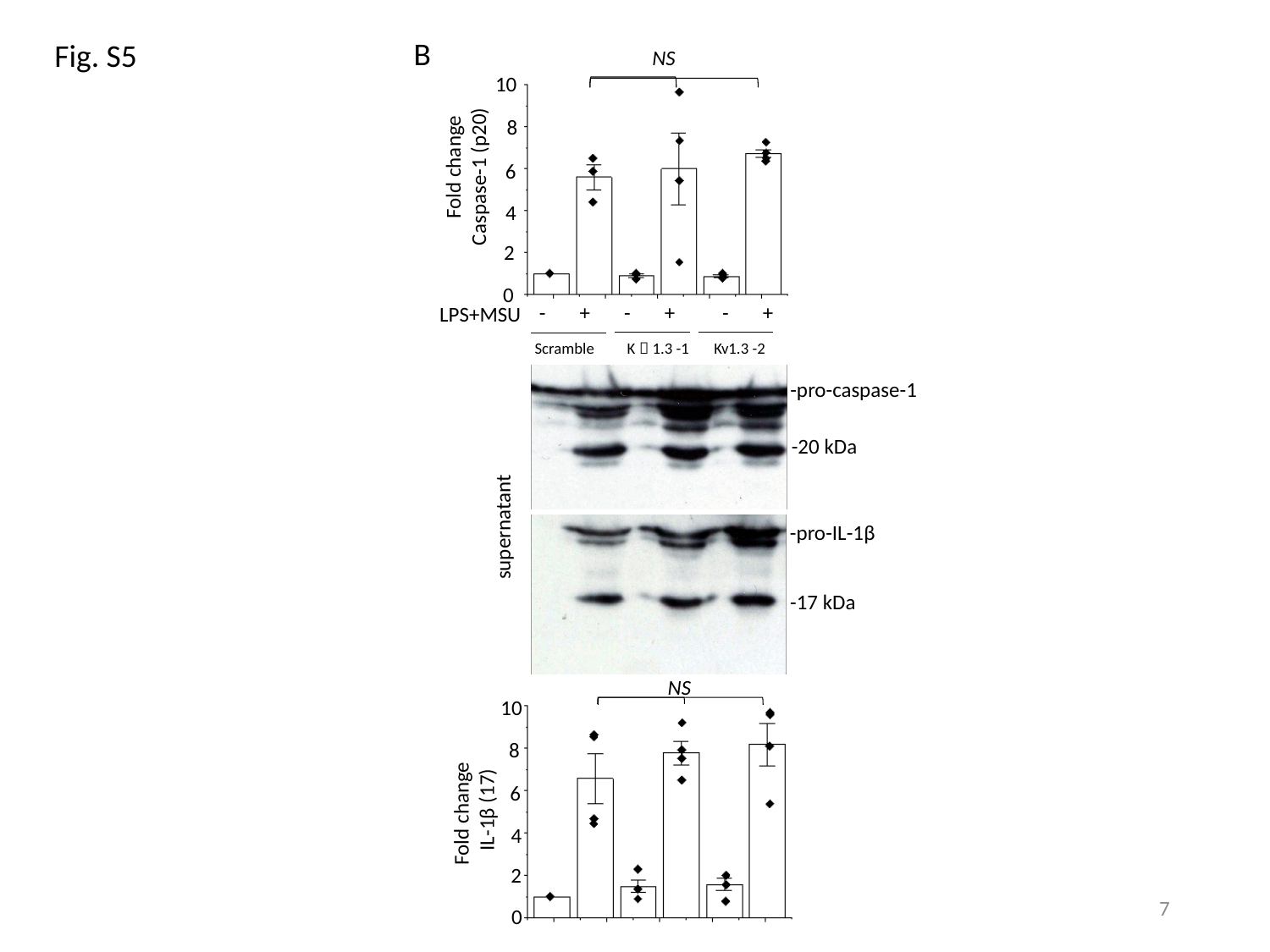

NS
10
　Fold change
Caspase-1 (p20)
8
6
4
2
0
B
- + - + 　 - +
LPS+MSU
Kｖ1.3 -1
Scramble
Kv1.3 -2
-pro-caspase-1
-20 kDa
-pro-IL-1β
-17 kDa
supernatant
NS
Fold change
 IL-1β (17)
10
8
6
4
2
0
Fig. S5
7

## Slide 8
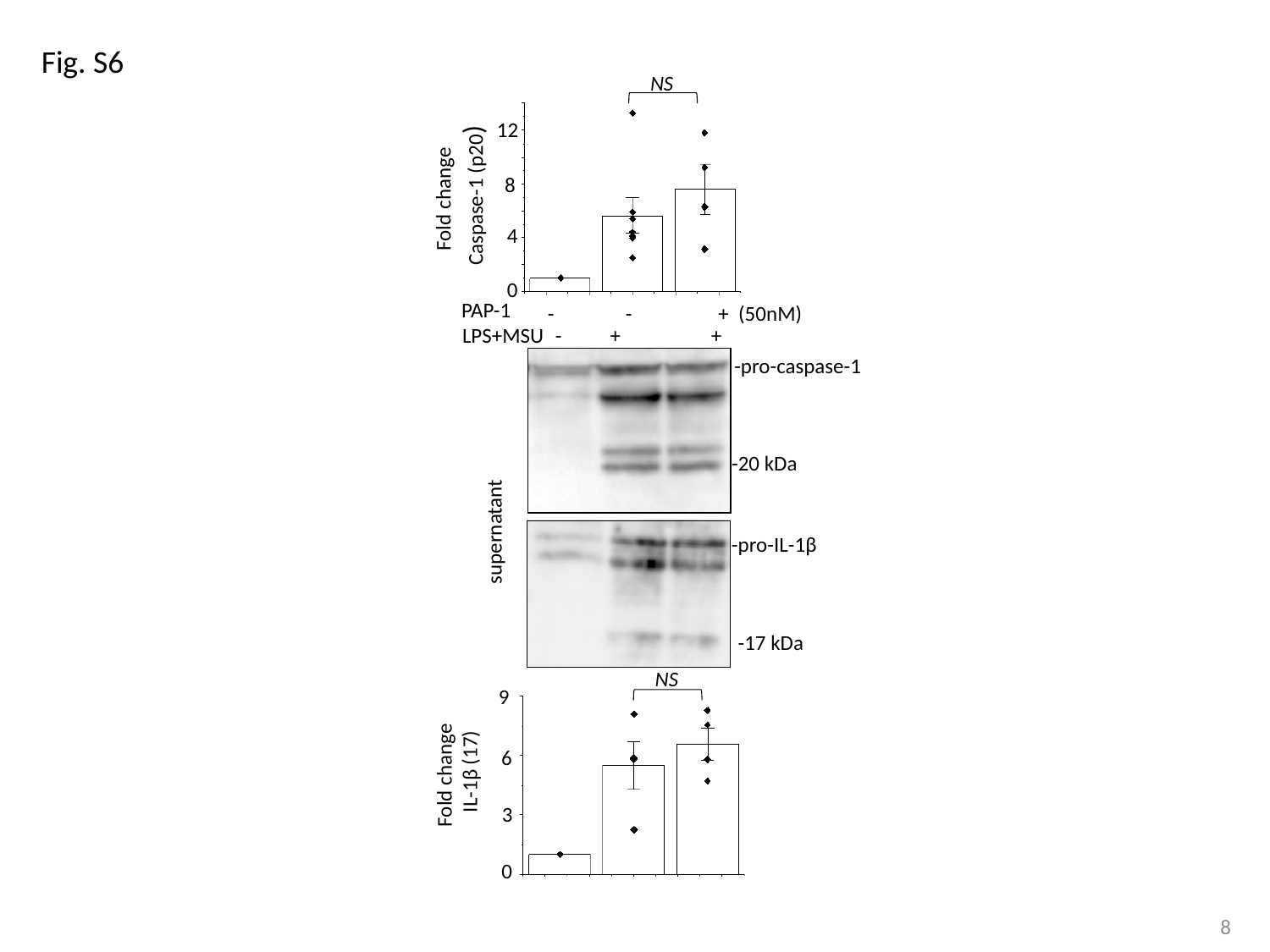

Fig. S6
NS
12
 Fold change
 Caspase-1 (p20)
8
4
0
PAP-1
 - 　 - 　 + (50nM)
LPS+MSU
 - + 　 +
-pro-caspase-1
-20 kDa
-pro-IL-1β
-17 kDa
supernatant
NS
9
6
Fold change
 IL-1β (17)
3
0
8

## Slide 9
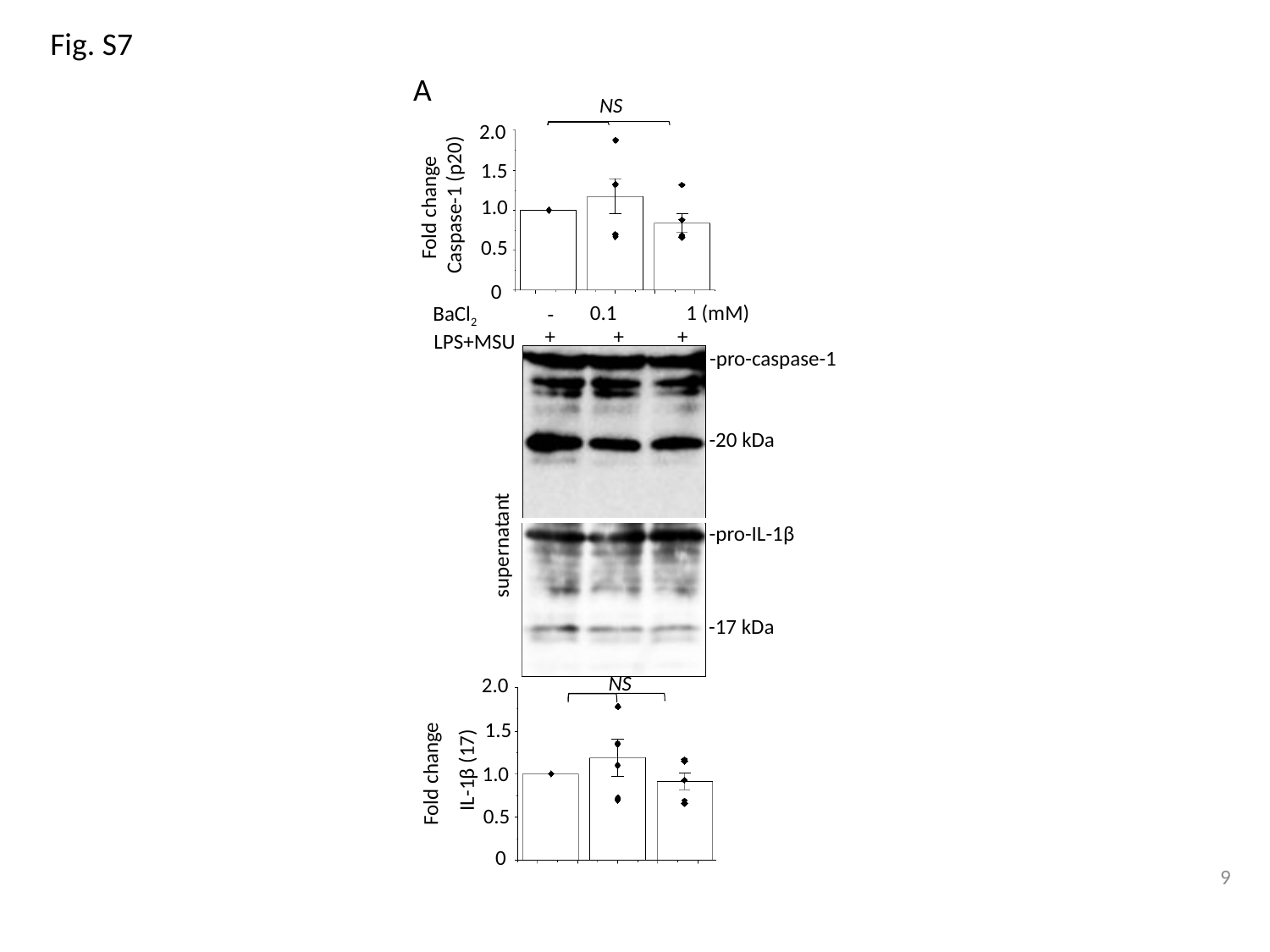

Fig. S7
A
NS
2.0
1.5
1.0
0.5
0
0.1 　 1 (mM)
BaCl2
-
+ + +
LPS+MSU
-pro-caspase-1
-20 kDa
supernatant
-pro-IL-1β
-17 kDa
NS
2.0
1.5
Fold change
 IL-1β (17)
1.0
0.5
0
 Fold change
 Caspase-1 (p20)
9

## Slide 10
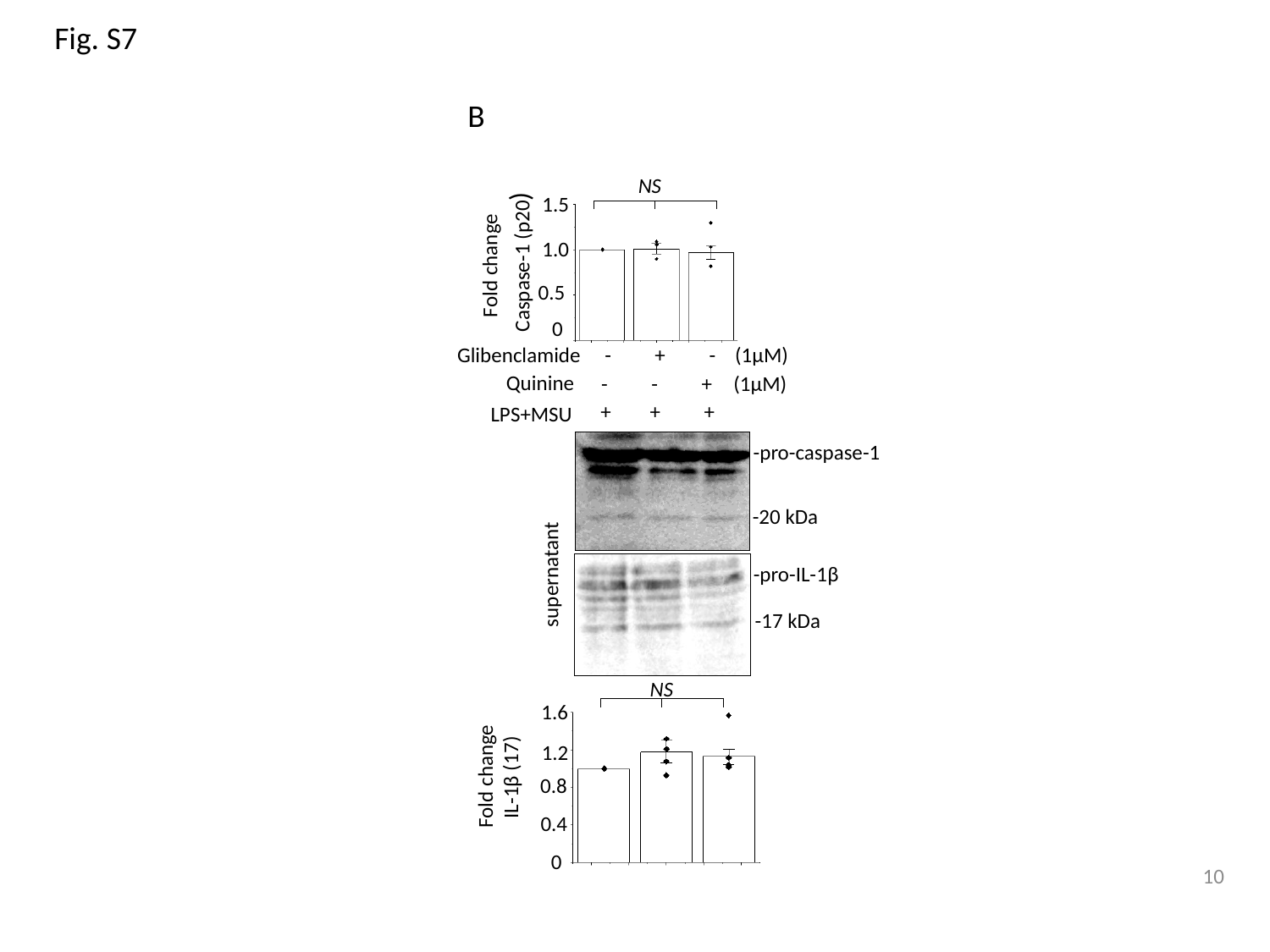

Fig. S7
B
 Fold change
 Caspase-1 (p20)
NS
1.5
1.0
0.5
0
Glibenclamide - + - (1μM)
Quinine
- - + (1μM)
+ + +
LPS+MSU
-pro-caspase-1
-20 kDa
-pro-IL-1β
supernatant
-17 kDa
NS
1.6
1.2
 Fold change
 IL-1β (17)
0.8
0.4
0
10

## Slide 11
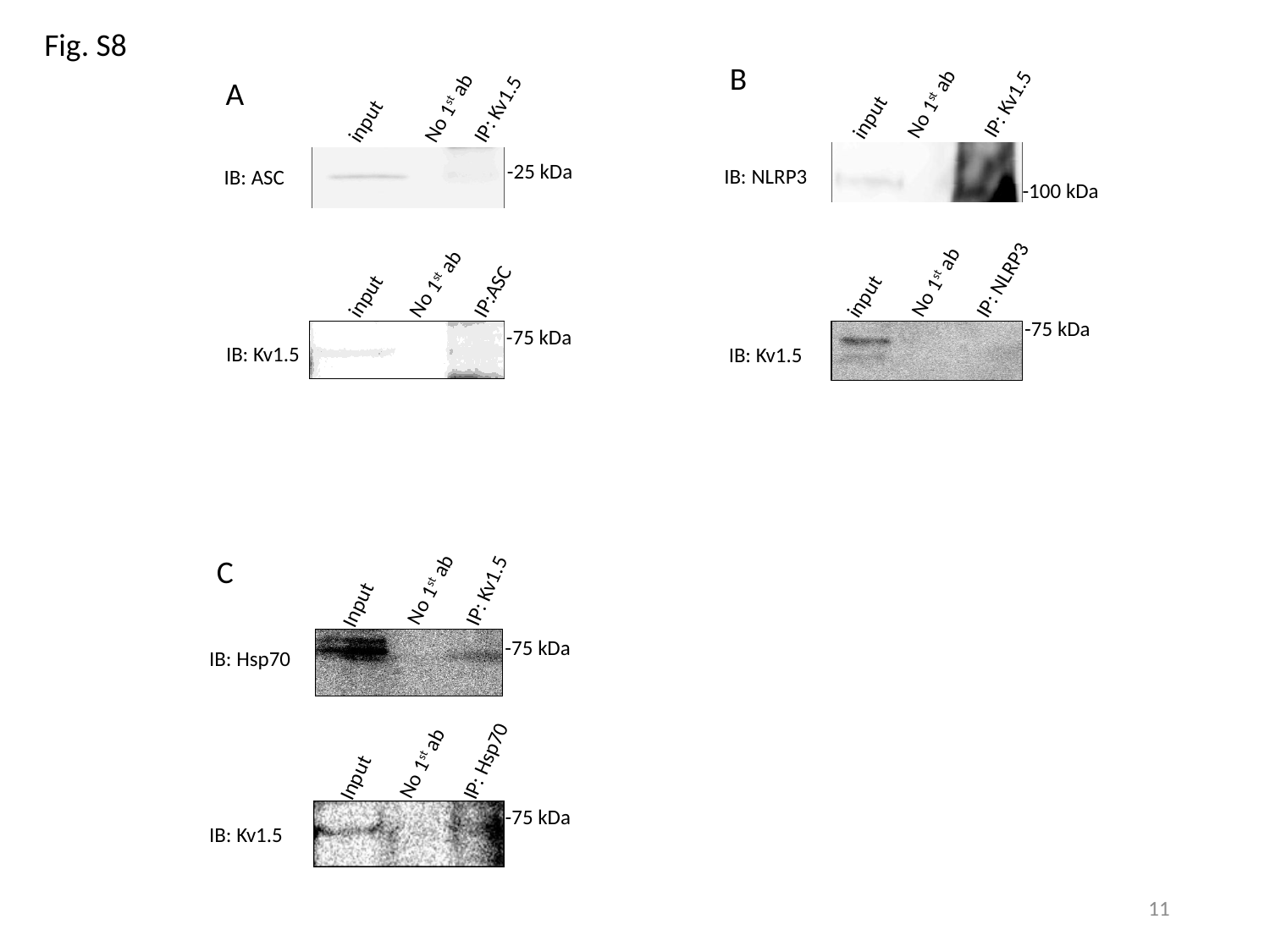

Fig. S8
B
No 1st ab
IP: Kv1.5
input
IB: NLRP3
-100 kDa
No 1st ab
IP: NLRP3
input
-75 kDa
IB: Kv1.5
No 1st ab
IP: Kv1.5
input
-25 kDa
IB: ASC
A
No 1st ab
IP:ASC
input
-75 kDa
IB: Kv1.5
No 1st ab
IP: Kv1.5
Input
-75 kDa
IB: Hsp70
C
Input
IP: Hsp70
No 1st ab
-75 kDa
IB: Kv1.5
11

## Slide 12
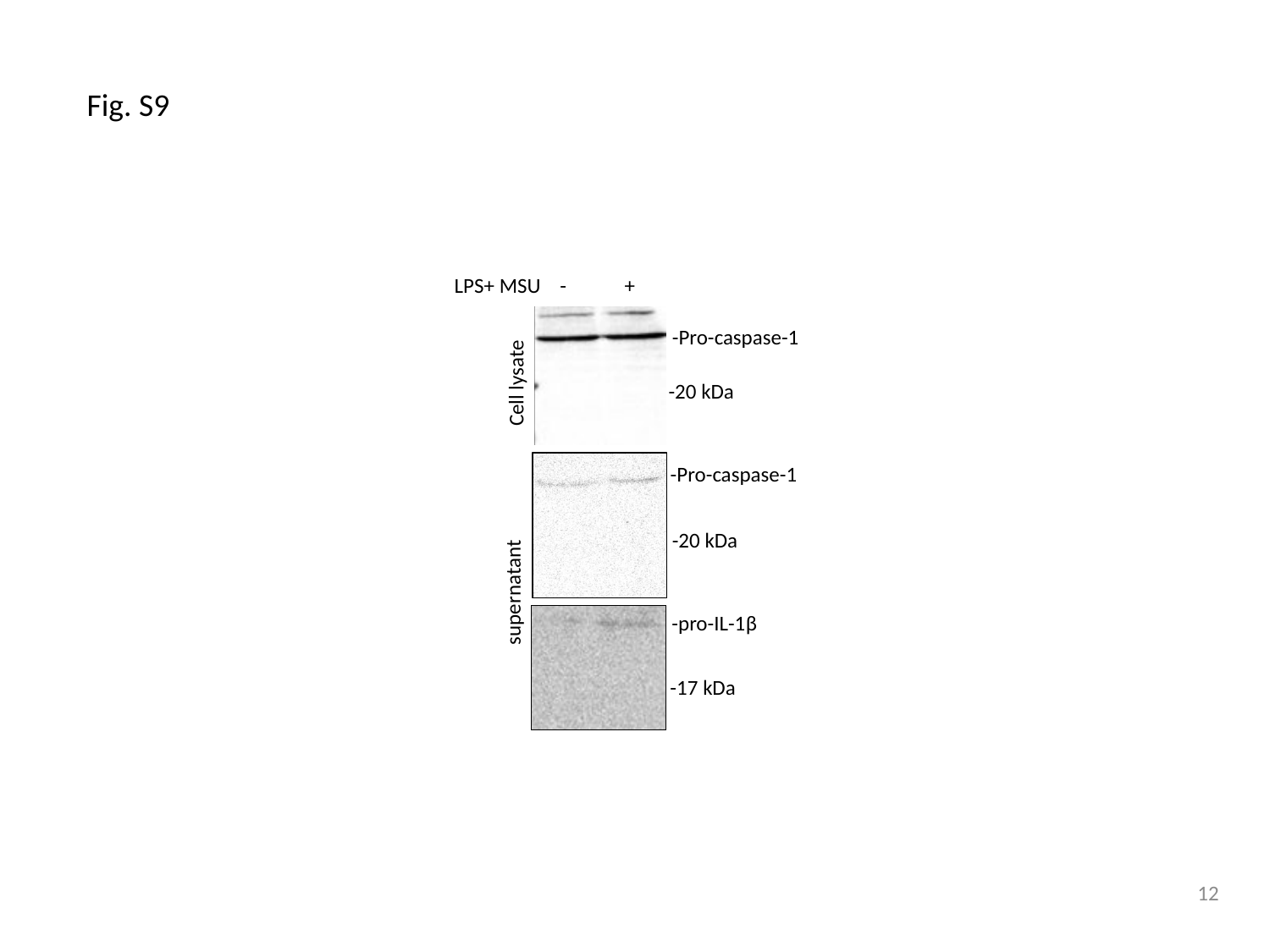

Fig. S9
LPS+ MSU - +
-Pro-caspase-1
Cell lysate
-20 kDa
-Pro-caspase-1
-20 kDa
supernatant
-pro-IL-1β
-17 kDa
12

## Slide 13
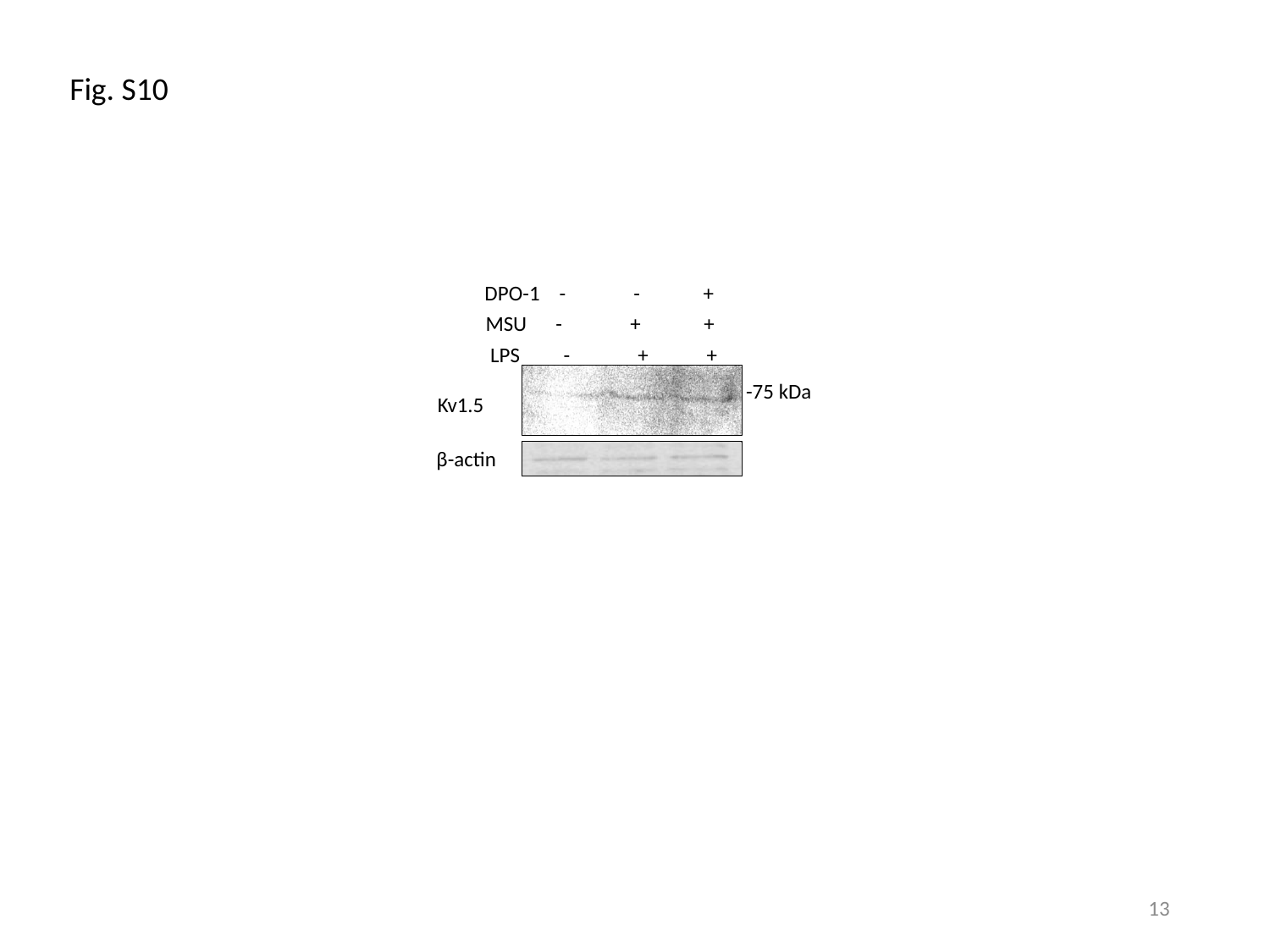

Fig. S10
DPO-1 - - +
MSU - + +
LPS - + +
-75 kDa
 Kv1.5
β-actin
13
